# Multiomics analysis reveals that chlorogenic acid alleviates heat stress-induced oxidative damage in prepubertal boar testes via the BLVRA-GPX3 pathway: in vivo and in vitro evidence

**DOI:** 10.1186/s40104-025-01336-0

**Published:** 2026-01-13

**Authors:** Shaoxuan Zhang, Dali Wang, Jiajia Qi, Jing Li, Simin Liu, Hao Sun, Shuang Liang, Boxing Sun

**Affiliations:** 1 Department of Animals Sciences, College of Animal Sciences, Jilin University, Changchun, 130062 China; 2 Poultry Institute, Shandong Academy of Agricultural Sciences, Jinan, 250000 China

**Keywords:** Chlorogenic acid, Heat stress, Multiomics, Oxidative damage, Testis

## Abstract

**Background:**

Heat stress (HS) can impair boar testicular function, leading to reproductive issues. However, chlorogenic acid (CGA) has been shown to mitigate HS-induced damage in various livestock and poultry species. Prepuberty is an important stage of testicular development in boars after birth. However, the protective effect of CGA on testicular HS injury during prepuberty boars and the underlying mechanisms are still not fully understood.

**Results:**

In vivo, a total of 30 healthy boars with similar body weights and ages were obtained and randomly divided into 3 groups, which were fed a basal diet supplemented with CGA 0 (the ND_TN group), 0 (the ND_HS group) or 1,000 (the CGA_HS group) mg/kg. After being fed for 28 d, all the groups, except the ND_TN group, were treated with high temperature for 7 d, after which samples were collected from the boars and analysed. The results showed that CGA significantly mitigated the HS-induced reduction in T-AOC content in testicular tissue and sperm density. Mechanistically, multiomics analysis revealed that the genes differentially expressed by CGA and HS were predominantly associated with the glutathione metabolism pathway. The combined analysis of transcriptomics and proteomics revealed that only *BLVRA* was affected by both HS and CGA when the mRNA and protein levels of a gene showed differential expression with the same trend. In vitro studies confirmed that CGA modulated GPX3 expression via *BLVRA*, affected GPx activity, and attenuated HS-induced ROS accumulation.

**Conclusions:**

In conclusion, prepubertal HS impairs the spermatogenic capacity of boars. *BLVRA* may mediate the testicular protective effect of CGA, although in vivo validation of this pathway is needed. This study contributes to elucidating the mechanisms underlying the effects of HS on prepubertal boar testicular development using multiomics approaches, laying a foundation for the potential utilization of CGA in swine production.

**Supplementary Information:**

The online version contains supplementary material available at 10.1186/s40104-025-01336-0.

## Introduction

Heat stress (HS) is the physiological response to elevated ambient temperatures and is exacerbated by factors such as global warming. The urgency of studying HS has been emphasized in light of climate change [1]. This condition leads to structural and functional impairments in various organ systems of mammals, impacting animal growth and development. Compared with female animals, most male mammals, in particular, are more sensitive to HS due to the unique feature of their testicles hanging out of the body [2]. The detrimental effects of HS on male mammals primarily include reduced feed intake, diminished daily weight gain, decreased libido or semen volume, and impaired sperm motility [3–5]. Male mammals are vulnerable to adverse effects of HS at different stages of life, including during foetal development and before and after sexual maturity [6–9]. However, there is a notable gap in research regarding the effects of HS on reproductive function during adolescence—a critical phase in male development, as highlighted by Ko [10]. *Sus scrofa,* commonly known as pigs, represents a valuable large animal model in biomedical research, offering advantages over smaller animals such as mice. Notably, compared with rodents, pigs exhibit Sertoli cell developmental timelines that more closely resemble those of humans [11, 12]. Moreover, the pig farming industry plays a significant role in Chinese agriculture. Nonetheless, the understanding of HS-induced testicular damage in prepubertal boars remains insufficient, necessitating further investigation.

Maintaining cellular oxidative homeostasis is crucial for normal cell function and overall health. HS primarily exerts its harmful effects through oxidative stress [13]. Several studies have indicated that HS disrupts testicular cell function and spermatogenesis via oxidative stress [9, 14, 15]. Previous studies have shown that the detrimental effects of HS can be mitigated by the external administration of antioxidants [16]. However, the selection and efficacy of antioxidants present complex challenges that necessitate further investigation.

Chlorogenic acid (CGA) is a bioactive compound that is abundant in *Euonymus ulmoides* leaves. It contains 5 hydroxyl groups and 1 carboxyl group. Its phenolic hydroxyl groups are highly reactive to free radicals and have strong antioxidant properties [17–20]. Our earlier study demonstrated that CGA protects testicular Sertoli cells from HS-induced damage in vitro [21]. Previous studies have shown that CGA promotes wound healing in diabetic rats without altering the levels of wound superoxide dismutase and catalase [22]. Additionally, CGA mitigates alcoholic liver injury by reducing the accumulation of oxidative products (-O^2^, ·OH, and H_2_O_2_) and suppressing oxidative stress [23]. Dietary supplementation with CGA enhances the antioxidant capacity of pork [24]. Based on the CGA supplementation doses reported in existing porcine studies [24–26], the dietary supplementation level of *Eucommia ulmoides* extract (containing 50% CGA) was set at 1,000 mg/kg in this experiment. We hypothesized that this dose might mitigate HS-induced testicular damage in prepubertal boars; however, further investigation is needed to clarify its actual efficacy and underlying molecular mechanisms.

A growing number of studies have applied different omics techniques to detect the effects of heat stress. A metabolomics analysis of muscle tissue from Nellore cattle utilizing ^1^H-NMR technology revealed that compared with low-precision animals, high-precision animals exhibited elevated levels of glutamine, betaine, creatinine, isoleucine, carnitine, and acetylcarnitine and decreased glucose levels [27]. Through proteomic sequencing of testicular tissue, researchers identified differentially abundant proteins influenced by VD3 that were enriched in pathways such as steroid hormone synthesis, steroid biosynthesis, peroxisome function, and fatty acid metabolism, which are closely associated with the regulation of testicular development [28]. Furthermore, through transcriptome sequencing, Yang et al. [29] reported that genes whose expression differed in Sertoli cells under HS conditions were enriched in pathways related to cellular stress response regulation, heat shock protein interactions, chaperone-mediated protein folding, sterol biosynthesis, and other processes. Traditional research methods, which are often confined to single-level analyses (e.g., individual genes, proteins, or metabolites), are insufficient to capture the complexity of biological systems. In contrast, multiomics technologies integrate transcriptomic, proteomic, and metabolomic datasets, enabling comprehensive, high-throughput, and rapid characterization of biomolecular dynamics [30, 31]. Previous studies have demonstrated that integrative multiomics analyses facilitate in-depth investigations of subtle biological responses to environmental and nutritional stimuli in animal models [32]. As a critical male reproductive organ, the testis exhibits precise and intricate self-regulatory mechanisms. Given this complexity, a single research approach may not fully capture the molecular perturbations within testicular tissues, thus, multiple complementary methods are required to dissect its regulatory networks comprehensively. Therefore, applying multi-omics approaches is essential to systematically elucidate the mechanisms by which HS and CGA affect the testes of prepubertal boars.

Given that testicular development in prepubertal animals directly affects adult boar reproductive function and based on prior research on CGA applications, we hypothesized that prepubertal boars exposed to HS would exhibit impaired postpubertal reproductive performance and that dietary CGA supplementation could enhance testicular antioxidant capacity in HS boars, thereby exerting a beneficial effect on spermatogenesis. In this study, we established an in vivo HS model in prepubertal boars and subjected Sertoli cells to HS in vitro. Using ^1^H-NMR, transcriptomics and proteomics, we aimed to comprehensively characterize changes in testicular metabolites, gene mRNA expression, and protein levels following heat stress and CGA treatment, along with their potential interrelationships. These analyses were performed to clarify the mechanism by which CGA alleviates HS-induced testicular damage in prepubertal boars. These findings will provide novel insights into the impact of HS on boar reproductive performance and contribute to advancing the theoretical research and application of CGA as a potential oxidative stress intervention to mitigate HS-related reproductive damage in boars.

## Materials and methods

### Experimental design for prepubertal boars

The prepubertal boars used in this study were from the Agricultural Experimental Base of Jilin University. A total of 30 approximately 6-week-old (39–44 days old, from 9 littered) Junmu No. 1 boars were weaned without castration. Their initial body weight was 10.11 ± 0.22 kg and they were randomly divided into three groups: the normal diet with thermoneutral (ND_TN) group, the normal diet with HS (ND_HS) group, and the CGA with HS (CGA_HS) group. There was no significant difference in body weight among the three groups (*P* > 0.05).

**Fig. 1:**
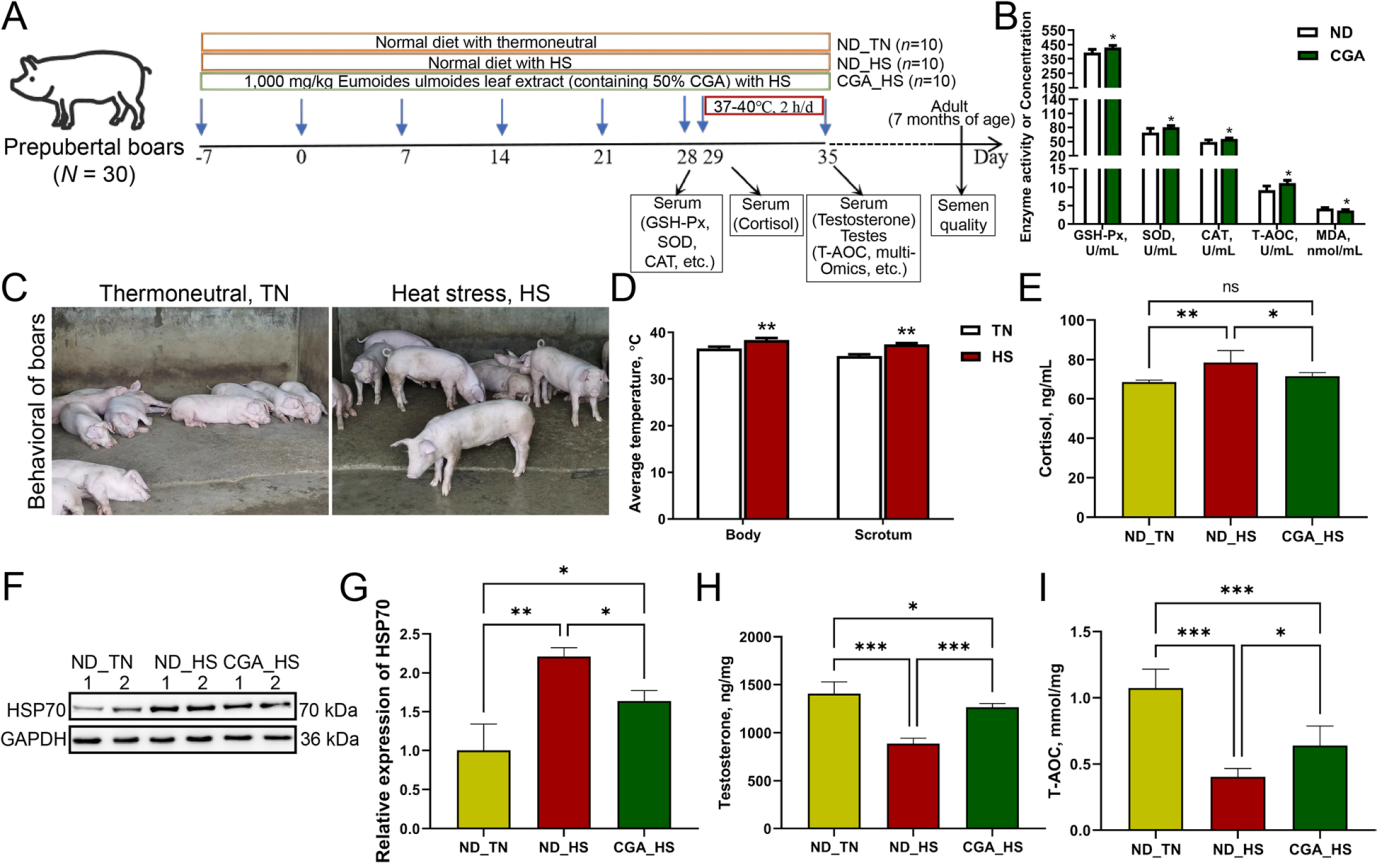
CGA affects the antioxidant capacity of heat-stressed boars. **A** Schematic of the experimental design. Black arrows indicate the days on which the samples were collected for analysis. **B** Contents of antioxidant indexes in the serum of prepubertal boars on D28 in the ND_HS and CGA_HS groups (mean ± SD,* n* = 10). **C** Representative images of behavioral performances of prepubertal boars under thermoneutral (TN) and heat stress (HS). **D** Body and scrotum temperatures of prepubertal boars under TN and HS ( *n* = 10). **E** Cortisol levels in the serum of prepubertal boars in different treatment groups on D29 (*n* = 10). **F** and **G** Protein levels and data statistics of HSP70 in the testes of prepubertal boars in different treatment groups on D35 (*n* = 5). **H** Testosterone in the serum of prepubertal boars in different treatment groups on D35 (*n* = 10). **I** T-AOC in the serum of prepubertal boars in different treatment groups on D35 (*n* = 5). Differences between two groups were analyzed using two-tailed Student's *t*-tests, and comparisons among multiple groups were performed using one-way ANOVA followed by Tukey’s multiple comparisons test. Data are expressed as the mean ± SD. Statistical significance was defined as *P* > 0.05 (ns), *P* < 0.05 (*), *P* < 0.01 (**), and *P* < 0.001 (***), indicating no, significant, highly significant, and extremely significant differences between two groups, respectively

The key time points and parameters measured are illustrated in Fig. 1A. The start of the formal trial was recorded as Day 1 (D1), and subsequent days were denoted accordingly. In reference to the amount of CGA added in a previous study [25], the CGA_HS group was supplemented with 1,000 mg/kg *Eumoides ulmoides* leaf extract (containing 50% CGA as determined by HPLC) purchased from Shanghe Biotechnology Co., Ltd. (Changsha, China). Boars in the ND_TN and ND_HS groups were fed a normal diet. In the CGA_HS groups, CGA was incorporated into the basal diet during a prefeeding period of one week (D−7 to D0) until the completion of heat treatment. Feed and freshwater were available ad libitum to all groups.

From D29 to D35 (7 consecutive days), boars in the ND_HS and CGA_HS groups were subjected to daily whole-body heat treatment at 37–40 °C for 2 h (12:00–14:00), with the ambient temperature maintained at 20–27 °C during non-treatment periods. This heat exposure protocol was adapted from a previously described method with minor modifications [33]. Boars in the ND_TN group were kept in a thermoneutral environment (20–27 °C) throughout the experimental period. During heat treatment, the body (neck region) and scrotal temperatures of all boars were measured at 13:00 using an infrared thermometer.

### Measurement of environmental temperature and humidity

Three electronic temperature-humidity meters were installed at 1.5 m above the ground. The selected installation locations should ensure good ventilation conditions, avoid direct sunlight and rain erosion, and be able to reflect the temperature and humidity conditions of the entire pigsty. Temperature and humidity data were recorded daily at 08:00, 13:00 and 18:00. The temperature-humidity index (THI) were formulated based on the reference [34]. Daily average THI was determined by averaging values from the three time points.

### Determination of growth performance in prepubertal boars

On D1, D28 and D35, the body weight of each pig was documented to calculate the average daily weight gain during each stage. Body weights of all piglets were measured after a 12-h fast at both the initiation and termination of the experiment. Throughout the trial period, total feed intake per group was recorded precisely, and the following growth performance parameters were calculated: average daily gain (ADG), average daily feed intake (ADFI), and feed-to-gain ratio (F/G).

### Determination of serum antioxidant indices, cortisol and testosterone levels

Serum samples collected on D28 were used to measure antioxidant indices, those collected on D29 were used to detect cortisol levels, and samples collected on D35 were employed to determine testosterone levels. Blood samples were obtained from the anterior vena cava and placed in procoagulant tubes. Following natural coagulation for 30 min, the whole blood was underwent centrifuged at 3,000 × *g* for 10 min to obtain serum. The levels of glutathione peroxidase (GSH-Px/GPx; HY-M0004), superoxide dismutase (SOD; HY-M0001), catalase (CAT; HY-M0018), total antioxidant capacity (T-AOC; HY-M0011) and malondialdehyde (MDA; HY-M0003) were detected according to the manufacturer's instructions (Beijing Sinouk Institute of Biological Technology, China). Serum cortisol and testosterone concentrations were quantified using enzyme-linked immunosorbent assay (ELISA) kits (cortisol: ml087204; testosterone: ml002339; Shanghai Enzyme-linked Biotechnology Co., Ltd., Shanghai, China) following the manufacturer’s protocols.

### Collection of prepuberty pig testicles

Following heat treatment on D35, five boars were randomly chosen from each group for weighing. After surgery, bilateral testicular tissue was excised, and testicular weight was measured postepididymis removal. The testicular index on D35 was determined as the ratio of testicular weight (g) to body weight (kg). A portion of the testicular tissue was promptly frozen in liquid nitrogen for total RNA and protein extraction, while another section was utilized for T-AOC assessment. A segment of the testicular tissue was preserved in Bouin's solution at 4 °C for 12 h for histological analysis, after which the remaining tissue was rapidly freozen in liquid nitrogen and storage at −80 °C.

### Total antioxidant capacity of boar testes

On the D35, fresh testicular tissue was homogenates to prepare tissue homogenate. After centrifugation, the supernatant was collected. The levels of T-AOC in testes were determined using Total Antioxidant Capacity Assay Kit with a Rapid ABTS method (S0121; Beyotime Biotechnology). All operations were performed following the manufacturer's instructions.

### HE staining of testicular tissue and counting of seminiferous tubule diameter and Sertoli cells

Testicular tissue paraffin sections were stained with haematoxylin and eosin for analysis. Thirty transverse sections of seminiferous tubules were examined per group to quantify the number of Sertoli cell. Following the methodology outlined by Ding et al. [35] and Franca et al. [36], seminiferous tubules with a difference of less than 5% between their two vertical diameters were selected as the subjects of observation. Sertoli cells are characterized by large nuclei, which are typically located in the basal layer of seminiferous tubules and exhibit triangular, irregular polygonal, or oval shapes with pale staining. The diameters of seminiferous tubules were measured in 10 randomly selected seminiferous tubules from the testicular sections of each boar, and the Sertoli cell count within each cross-section of the seminiferous tubules was recorded.

### Semen quality analysis after sexual maturation

The remaining five boars in each group were housed in 20–27 °C pens until 7 months of age, at which point the semen samples were collected and analysed. Semen was collected via the gloved-hand method. Each boar was ejaculated three times with a 3-d interval between collections. Ejaculate volume was analysed by weight in grams. For analysis, 1 μL of semen was mixed with 9 μL of diluent, and a 10-μL aliquot was placed on a glass slide. Sperm density, viability, motility and kinematic parameters were measured using the Weili Color Sperm Quality Detection System (Model WLJY9000) with three technical replicates.

### Extraction of testis metabolites for ^1^H-NMR-based metabolomics assays

Testicular samples were processed using one-dimensional nuclear magnetic resonance hydrogen spectroscopy (^1^H-NMR) following established protocols [37]. Briefly, testicular tissues were centrifuged, extracted, and dissolved, and the levels of water-soluble metabolites were determined by ^1^H-NMR. Analysis was conducted using an AVANCE III 600 MHz NMR spectrometer at 298 K. The free induction decay (FID) signal from the ^1^H-NMR was processed using MestReNova (Mnova 12.0) software, and the NMR spectra were calibrated with TSP (ppm 0.00). Fourier transform, phase correction and baseline correction were performed automatically. The integration window ranged from ppm 0.4 to 9.6, with the exclusion of the 4.62–5.2 ppm range to eliminate residual water peak interference, using an interval δ0.005. Normalization of the spectra to a total area of 100 was conducted for subsequent analysis. Metabolite names were assigned based on entries in the Human Metabolome Database (HMDB, https://hmdb.ca/) and relevant literature sources [38–42]. Data normalization was performed using SIMCA-14.1 software from Umetrics.

### Correlation analysis between metabolites and boar traits

Bivariate correlations were computed utilizing Spearman’s Rank coefficients. Heatmaps were generated through the NovoMagic cloud platform (https://magic-plus.Novogene.com).

### Testis RNA extraction, cDNA preparation and transcriptome data analysis

Total RNA was extracted from the testicular tissues using RNAiso Plus reagent (9108; Takara, Kyoto, Japan), followed by quantification using an ultraviolet spectrophotometer. RNA integrity was assessed using an Agilent 540 bioanalyzer. High-quality RNA samples were stored at −80 °C for subsequent library preparation using the NEBNext^®^ Ultra™ RNA Library Prep Kit for Illumina^®^. The prepared libraries were sequenced on an Illumina NovaSeq 600 platform (Illumina, USA) following the “sequencing by synthesis” method, generating 150 bp paired-end reads (Beijing, China). Differential expression analysis was performed using the DESeq2 R package (1.20.0). The resulting *P* values were adjusted using the Benjamini and Hochberg’s approach for controlling the false discovery rate (FDR). Differentially expressed genes (DEGs) were identified based on a significance threshold of *P*-value < 0.05 and |log_2_fold change| ≥ 0.36. Gene Ontology (GO) and Kyoto Encyclopedia of Genes and Genomes (KEGG) enrichment analyses were subsequently conducted on the identified genes and transcripts. The heatmap depicts hierarchical clustering (H-cluster) of log_2_-transformed (FPKM + 1) and z-score-normalized expression levels of genes.

### Total protein extraction and proteomic analysis of testicular tissues

Tandem mass tag (TMT) technology was employed for proteomic sequencing analysis. Briefly, approximately 100 mg of frozen testicular tissue samples were retrieved from −80 °C, ground into a powder at low temperature, and subjected to protein extraction using PASP cleavage. A Bradford protein quantification kit was used to determine the protein concentration, and polyacrylamide gel electrophoresis (SDS-PAGE) was used to assess protein integrity and purity. The qualified proteins were subsequently labelled with TMT. Proteins were identified against the 994935-Ensamble-20211126-Sus_scrofa.Sscrofa11.1….fasta. Raw data were generated following the enrichment of modified peptides (suitable for modified proteomes), and fractionation was then performed against the corresponding database to identify proteins and perform mass spectrometry analysis. The raw files obtained from mass spectrometry were then queried against the corresponding database to identify proteins. Concurrently, a mass tolerance distribution analysis of the peptide, protein and parent ion was performed to assess the quality of the mass spectrometry detection data. Subsequent protein quantitative analysis included overall differential protein analysis, differential protein screening, and expression pattern cluster analysis. To improve the quality of analysis results, the software PD 2.2 further filtered the retrieval results: Peptide Spectrum Matches (PSMs) with a credibility of more than 99% were identified as PSMs. The identified protein contains at least 1 unique peptide. The identified PSMs and proteins were retained, and the FDRs were no more than 1.0%. Proteins with a corrected *P*-value < 0.05 and a fold change > 1.2 or < 0.83 were considered significantly differentially expressed. A total of 7,380 proteins were identified across all the samples. Finally, the differentially expressed proteins were subjected to GO and KEGG functional enrichment analyses.

### Combined analysis of transcriptomics and proteomics

The primary analyses included association studies of gene and protein expression and GO functional enrichment analysis. A significance threshold of *P*-value < 0.05 was employed to identify statistically significant differences in the analyses.

### In vitro cell culture and processing

Sertoli cells (ATCC^®^ CRL-1746) were treated with HS and CGA in accordance with established protocols [21]. siRNAs targeting BLVRA were synthesized by RiboBio Company (Guangzhou, China), and the target sequences are detailed in Table S1. siRNAs transfection was performed using Lipofectamine™ 2000 (11668030; Thermo Fisher, USA) following the manufacturer's guidelines. After 24 h of transfection, CGA was added, followed by heat stress treatment after 24 h of incubation.

### Total RNA extraction of cells and RT-qPCR

Total RNA was isolated from cells employing RNAiso Plus reagent (9108; Takara, Kyoto, Japan), after which the RNA concentration and purity were assessed using a spectrophotometer (NanoDrop 2000c; Thermo Scientific, USA). cDNA was subsequently synthesized via reverse transcription using the PrimeScript™ RT Reagent Kit with gDNA Eraser (RR047A; Takara) following the manufacturer’s protocol. RT-qPCR was conducted on a CFX Duet Real-Time PCR System (Bio-Rad, USA) using SYBR Green Master Mix (0491914001; Roche) with three technical replicates. The sequences of primers used for RT-qPCR were designed using Primer Premier 6 software, as detailed in Table S2. Relative mRNA expression levels were quantified using the 2^−^^ΔΔCt^ method and normalized to those of *GAPDH*.

### Total protein extraction of cells and Western blot

Cellular protein extraction and Western blot analysis were conducted as per the established protocol [21] with minor adjustments. In brief, following the Western chemiluminescence assay, the samples were washed in distilled water for 5 min, after which an appropriate volume of Western primary and secondary antibody removal solution (P0025B; Beyotime Biotechnology, China) was added. The samples were then agitated on a shaker for 10 min, after which the solution was discarded. The PVDF membrane was rinsed three times with TBS for 3 min each on a shaker. Subsequent steps included Western blot blocking and additional procedures.

### Detection of intracellular ROS levels

Intracellular reactive oxygen species (ROS) were detected following the protocol of the Reactive Oxygen Species Assay Kit (S0033; Beyotime Biotechnology, China) with three technical replicates. In brief, a solution of serum-free medium and DCFH-DA at a 100:1 ratio, resulting in a final concentration of 10 μmol/L, was prepared. Upon reaching the specified incubation time in 6-well plates, the culture medium was aspirated, and each well was treated with 1 mL of the prepared solution. Additionally, 1 μL of 50 mg/mL Rosup was added exclusively to the positive control well. The cells were subsequently incubated at 37 °C for 20 min. After this, the cells were washed three times with serum-free medium, and fluorescence images were captured using excitation/emission wavelengths of 488 nm/525 nm. ImageJ software was used for the analysis of fluorescence images to determine fluorescence values, which are indicative of intracellular ROS levels.

### Detection of total intracellular glutathione peroxidase

Total glutathione peroxidase (GPx) activity was detected following the instructions of the Total Glutathione Peroxidase Assay Kit with NADPH (S0058; Beyotime Biotechnology, China) with three technical replicates. Briefly, at the end of cell treatment in 6-well plates, the cells were rinsed with PBS, lysed, and centrifuged at 12,000 × *g* for 10 min at 4 °C. The supernatant was used for enzyme activity assays. Subsequently, in a 96-well plate, a sequence of detection buffer, test sample, and GSH-px detection working solution was added and mixed. Following the addition of 40 μL of the Gpx detection working solution, the mixture was incubated at room temperature for 15 min. Subsequently, 10 μL of 30 mmol/L peroxide reagent solution was added to each well and mixed. The optical density (OD) at 340 nm was promptly determined using a microplate reader, with the initial reading recorded as the 0 min value. OD values at 340 nm were then recorded at 1 min intervals for 5 consecutive time points, resulting in a total of 6 data points. The GPx activity in the samples was calculated based on a constructed standard curve.

### Measurement of cell viability

Sertoli cells were cultured in 96-well plates. CCK-8 method (K1018; ApexBio Technology) was used to determine cell viability, and the specific procedures were carried out according to the manufacturer's instructions. Briefly, at the end of the cell treatment, 10 μL of CCK-8 solution was added to each well and incubated in an incubator at 37 °C for 1 h. The absorbance at 450 nm was measured with a microplate reader (Bio-Rad, USA). The OD values were used for statistical analysis of cell viability in this study.

### Statistical analysis

All the data were analysed using SPSS 19.0 software (IBM Corp., Armonk, NY, USA). Two-tailed Student's *t*-tests were used to compare differences between two groups, whereas one-way ANOVA was used for comparisons among multiple groups. The results are presented as the mean ± SD. Significance was set at *P* > 0.05, denoted as (ns); *P* < 0.05, denoted as (*); *P* < 0.01, denoted as (**); and *P* < 0.001, denoted as (***), indicating no significant, significant, highly significant, and extremely significant differences between the treatment and control groups, respectively. 

## Results

### Effects of the CGA on boars and construction of an HS model of prepuberty boars

The antioxidant effect of CGA was evaluated by measuring the activities of GSH-Px, SOD, CAT, and T-AOC and the content of MDA in serum on D28. The results are shown in Fig. 1B. The activities of GSH-Px, SOD, CAT and T-AOC significantly increased in the CGA group (*P* < 0.05), whereas the content of MDA was significantly decreased (*P* < 0.05). The effects of HS and CGA on the growth performance of prepuberty boars are shown in Table S3. The feed-to-gain ratio (F/G) of boars in the ND_HS group during heat treatment was were significantly higher than that in the ND_TN group (D29–D35; *P* < 0.05). The final body weight and average daily gain of boars in the CGA_HS group were significantly greater than those in the ND_TN and ND_HS groups (D1–D35; *P* < 0.05).

During the heat treatment, the boars exposed to the suitable environment (TN, 20–27 °C) were mostly quiescent, while boars exposed to the heat treatment (HS, 37–40 °C) were agitated and had increased water consumption (Fig. 1C). Moreover, the body surface temperature and scrotal epidermal temperature of the boars in the heat treatment group were significantly greater than those in the non-heat treatment group (*P* < 0.01; Fig. 1D). The results of serum cortisol content detection are shown in Fig. 1E. Compared with the ND_TN group, HS significantly increased the serum cortisol content in the ND_HS group (*P*  < 0.01). In comparison with the ND_HS group, CGA significantly reduced the serum cortisol content in the CGA_HS group (*P*  < 0.05). As shown in Fig. 1F and G, the HSP70 of the ND_HS group was significantly greater than that of the ND_TN group (*P*  < 0.01). Compared with ND_HS, the CGA_HS significantly decreased the expression of HSP70 (*P*  < 0.05). As shown in Table S4, during the experimental period, the THI values at 13:00, 18:00, and the daily average THI were significantly higher in the ND_HS and CGA_HS groups than in the control and ND_TN groups (*P* < 0.05). These findings indicated that the HS model of prepuberty boars was successfully established in this experiment.

The results of serum testosterone level detection on D35 showed that HS significantly induced a decrease in testosterone level (*P* < 0.001). Compared with the ND_HS group, CGA in the CGA_HS group significantly increased the testosterone level (*P* < 0.001; Fig. 1H). Next, we examined the total antioxidant capacity of testicular tissue at D35, as shown in Fig. 1I, where CGA restored the reduced T-AOC content due to HS (*P* < 0.05).

### HS in prepubertal boars affects sperm density after their sexual maturity

**Fig. 2:**
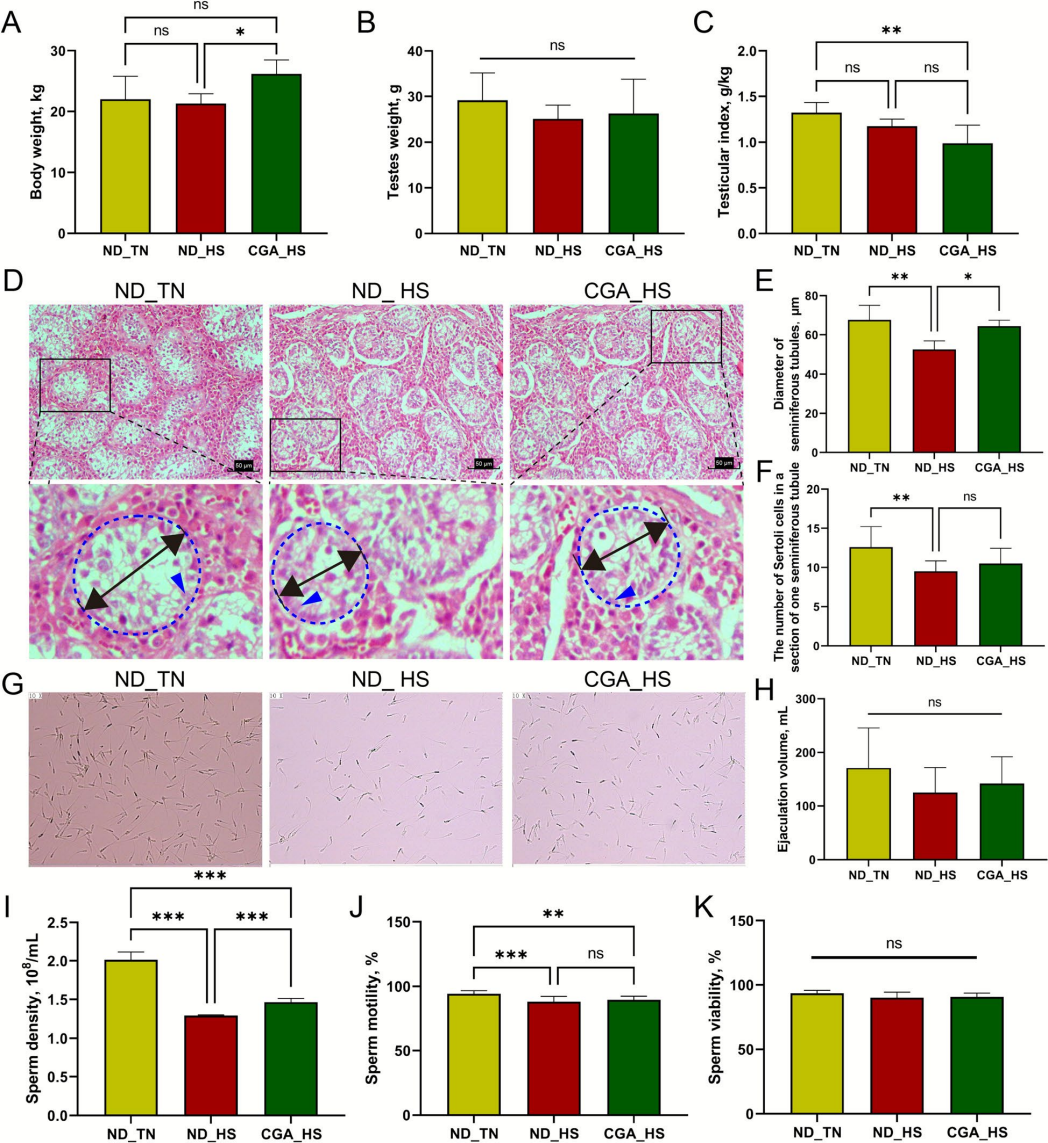
Effects of HS and CGA on testicular development in prepubertal boars and subsequent semen quality post-sexual maturity. **A** Body weights of pre-pubertal boars at D35 with testicular tissue collection in different treatment groups (*n* = 5). **B** Testis weight of pre-pubertal boars at D35 with testicular tissue collection in different treatment groups (*n* = 5). **C** Testicular index of pre-pubertal boars at D35 with testicular tissue collection in different treatment groups (*n* = 5). **D** Representative images of the testes by HE staining (400 ×; top row). The bottom row shows magnified views of the areas outlined by solid black rectangles in the top row, where dashed blue lines indicate the contours of seminiferous tubules, black double-headed arrows denote seminiferous tubule diameters, and blue arrows point to Sertoli cells. **E** Seminiferous tubule diameters of pre-pubertal boars in different treatment groups (*n* = 5). **F** Representative images of semen quality assessment in sexually mature boars from different groups (10 ×). **G** The number of Sertoli cells in a cross section of seminiferous tubules (*n* = 5). **H** The ejaculate volume of pre-pubertal boars in different treatment groups (*n* = 5). **I** The sperm density of pre-pubertal boars in different treatment groups (*n* = 5). **J** The sperm motility of pre-pubertal boars in different treatment groups (*n* = 5). **K** The sperm viability of pre-pubertal boars in different treatment groups (*n* = 5). Data are expressed as the mean ± SD. ^ns^*P* > 0.05, ^*^P < 0.05, ^**^P < 0.01, ^***^P < 0.001

As shown in Fig. 2A, the body weights of the ND_HS group were lower than those of the CGA_HS group (*P* < 0.05). No significant difference in testis weight was observed among the three groups (*P* > 0.05; Fig. 2B). However, the testicular index in the CGA_HS group was significantly lower than that in the ND_TN group (*P* < 0.01; Fig. 2C).

Next, HE staining was used to observe the seminiferous tubules in testicular tissues after different treatments (Fig. 2D). The diameter of seminiferous tubules and the number of Sertoli cells within them were analysed (Fig. 2E and F). HS significantly reduced the diameter of seminiferous tubules and decreased the number of Sertoli cells in the testis (*P* < 0.01), while CGA increased the diameter of seminiferous tubules (*P* < 0.05) but had no significant effect on the number of Sertoli cells (*P* > 0.05).

To investigate whether short-term HS in boars at prepuberty affects spermatogenesis, we analysed the semen quality of male boars after sexual maturity. The results shown in Fig. 2H and K showed no significant differences in ejaculate volume or sperm viability among all groups (*P* > 0.05). Compared with the ND_TN group, the sperm density (Fig. 2I) and sperm motility (Fig. 2J) in the ND_HS group were significantly decreased (*P* < 0.001). Additionally, the sperm density in the CGA_HS group was significantly higher than that in the ND_HS group (*P* < 0.05). No significant differences were observed in sperm kinematic parameters among all groups (Fig. S1).

### Metabolites in testicular tissue affected by HS and CGA via ^1^H-NMR

**Fig. 3:**
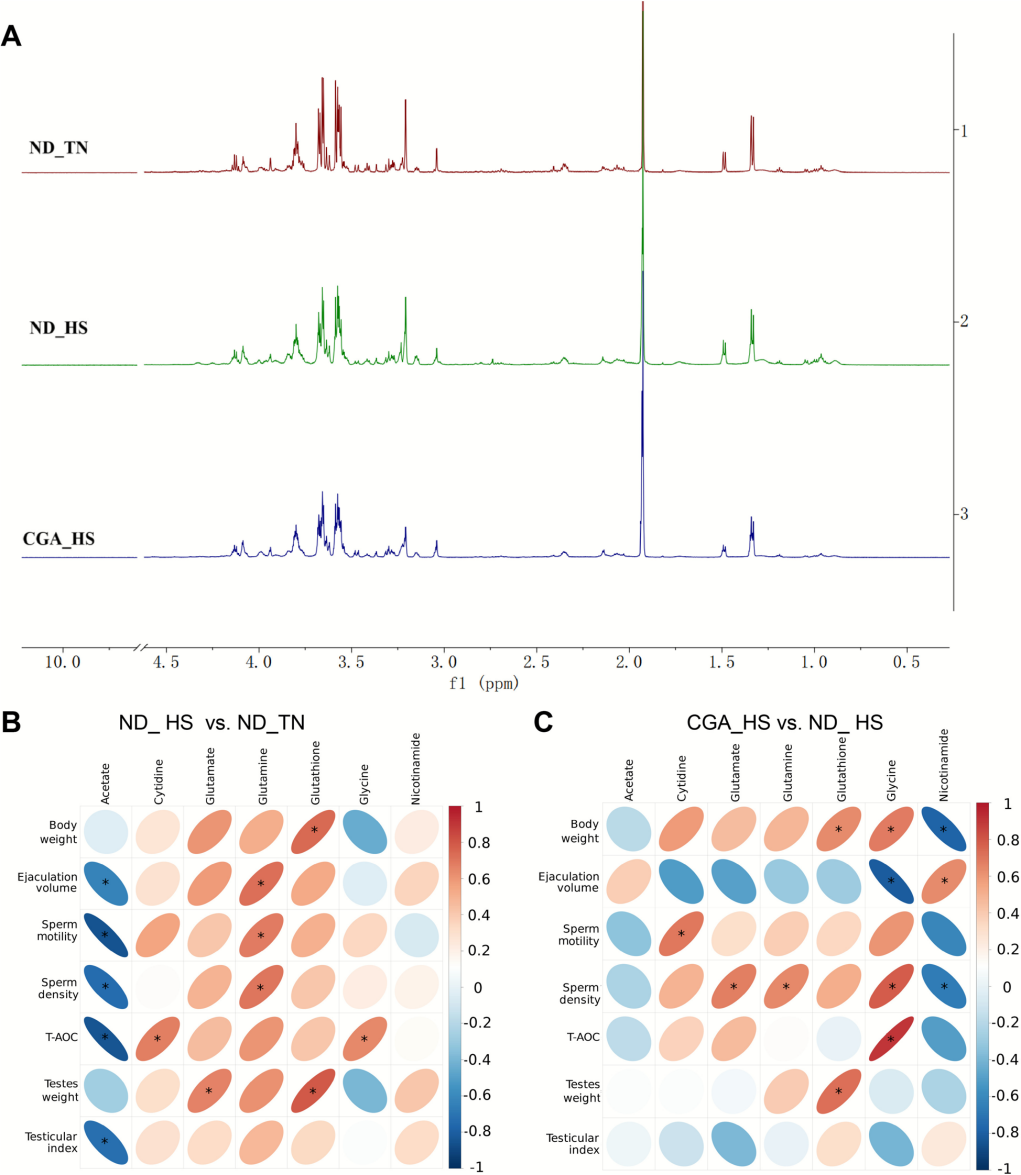
Effects of HS and CGA on metabolites in the testes of heat-stressed pigs determined by ^1^H-NMR methods. **A**^1^H-NMR superposition (0–10 ppm) of testicular tissue in ND_TN, ND_HS and CGA_HS group. **B** Heatmap of the correlation between 7 animal traits and 7 metabolites in ND_HS vs. ND_TN. **C** Heatmap of the correlation between 7 animal traits and 7 metabolites in CGA_HS vs. ND_HS. Each column corresponds to a trait of boars, and each row corresponds to a metabolite. The color of the oval is the correlation coefficient, red (R > 0) is the positive correlation, blue (R < 0) is the negative correlation, the area of the oval is the *P* value, and the * indicates the *P* value < 0.05

Changes in small molecule metabolites constitute the visual embodiment of the final phenotype of the body in response to external changes. Therefore, to elucidate the changes in the body during the process through which CGA relieves HS in testicular tissue more directly, we conducted metabolomic sequencing. We identified 35 metabolites from testicular water-soluble extracts using ^1^H-NMR (Fig. 3A, Fig. S2 and Table S5). To further investigate the effects of CGA and heat treatment on testicular metabolites, we calculated the relative content of the aqueous metabolites and statistical significance, as shown in Table S6. Compared with those in the ND_TN group, the contents of acetate in the ND_HS group were significantly increased, while the contents of glutamate, glutamine and glutathione significantly decreased (*P* < 0.05). Compared with those in the ND_HS group, the contents of glycine and gytidine were significantly increased, and the content of nicotinamide was significantly decreased in the CGA_HS group (*P* < 0.05).

Next, we used correlation heatmaps to analyse 7 animal shapes (prepubertal: body weight, testis weight and testis coefficient and testicular T-AOC; after sexual maturity: ejaculate volume, sperm density, and sperm motility) and 7 metabolites (glutamate, glutamine, glutathione, glycine, nicotinamide and cytidine) in the testes of prepubertal boars (Fig. 3B and C). The results revealed that in the ND_HS and ND_TN groups, acetate was significant negative correlations with ejaculation volume, sperm motility, T-AOC and testicular index (*P* < 0.05); moreover, cytidine was significantly positively correlated with T-AOC (*P* < 0.05), and glutamate was significant positive correlations with testicular weight (*P* < 0.05). Glutamine was positively correlated with ejaculate volume, sperm motility, and post-maturation sperm density (*P* < 0.05). Glutathione displayed positive correlations with body weight and testicular weight (*P* < 0.05), and glycine was significantly positively correlated with testicular T-AOC (*P* < 0.05). In the CGA_HS vs. ND_HS comparison group, the correlation patterns of three metabolites were consistent with those observed in the ND_HS vs. ND_TN group, which glutathione maintained positive correlations with body weight and testicular weight; glutamine remained positively correlated with sperm density, and glycine retained a positive correlation with testicular T-AOC. The correlation coefficients and *P* values are shown in Table S7.

### RNA-seq analysis of changes in transcript expression in testes after exposure to HS and CGA

**Fig. 4:**
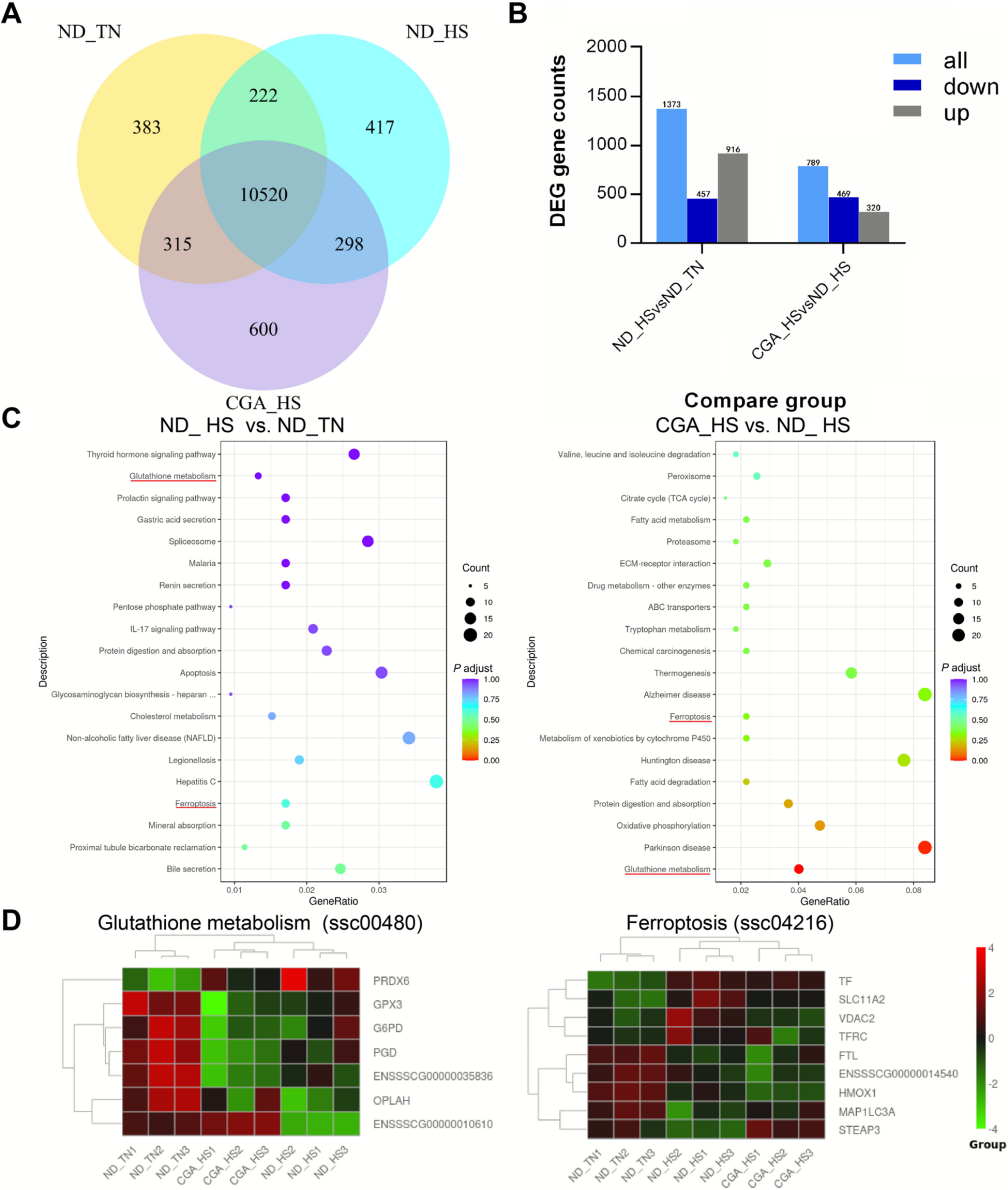
Impact of HS and CGA on testicular transcriptome in prepubertal boars. **A** Veen map of coexpressed genes in the ND_TN, ND_HS and CGA_HS groups. The overlapping numbers indicate the count of genes co-expressed between two or three groups. **B** The number of DEGs in the ND_TN vs. ND_HS and ND_HS vs. CGA_HS groups. **C** The enrichment analysis map of KEGG of ND_TN vs. ND_HS (left) and ND_HS vs. CGA_HS (right). The scatter plot illustrates KEGG pathway enrichment of DEGs. The *x*-axis represents the ratio of genes in the target pathway to the total number of genes in the gene list. The *y*-axis displays top enriched pathway terms. Dot color corresponds to the *P*_adj_, and dot size reflects the number of DEGs annotated to each pathway term. **D** Heatmap of EDGs involved in glutathione metabolism (left) and ferroptosis (right) as detected by RNA-seq. The *x*-axis denotes sample names (*n* = 3), and the *y*-axis represents gene identifiers. Red indicates a higher level; green indicates a lower level

To understand the molecular mechanism through which CGA affects testicular HS, RNA-seq was performed. As shown in Fig. 4A, there were 10,520 coexpressed genes in the three groups. In addition, 1,373 genes were differentially expressed between ND_HS and ND_TN, including 457 upregulated genes and 916 downregulated genes. A total of 789 DEGs were identified between CGA_HS and ND_HS, of which 469 were upregulated and 320 were downregulated (Fig. 4B and Table S8).

To verify the accuracy of the sequencing results, mRNA changes in the 8 DEGs were detected by RT-qPCR. The results are shown in Fig. S2A and S2B, where Fig. S2A shows the FPKM values of 8 genes from the transcriptome sequencing data and Fig. S2B shows the relative expression of each gene from the quantitative results. The results revealed that the variation trend of the 8 genes was essentially consistent with that of transcriptome sequencing results, indicating that the sequencing results were accurate and could be used for further research.

KEGG enrichment analysis of DEGs are shown in Fig. 4C. The results showed that the DEGs in ND_HS vs. ND_TN were enriched in glutathione metabolism (ssc00480), oxidative phosphorylation (ssc00190), protein degradation and absorption (ssc04974) and thermogenesis (ssc04714). The DEGs between CGA_HS and ND_HS groups were significantly enriched in glutathione metabolism (ssc00480) (*P* < 0.05). The DEGs in the two comparison groups were enriched in glutathione metabolism and ferroptosis pathways. The corresponding gene expression heatmaps are shown in Fig. 4D, and gene expression data are provided in the Tables S9 and S10. As shown in Fig. 4D, 7 DEGs, including *PRDX6* and *GPX3*, were enriched in the glutathione metabolic pathway, while 9 DEGs, including *TF* and *SLC11A2*, were enriched in the ferroptosis pathway.

**Fig. 5:**
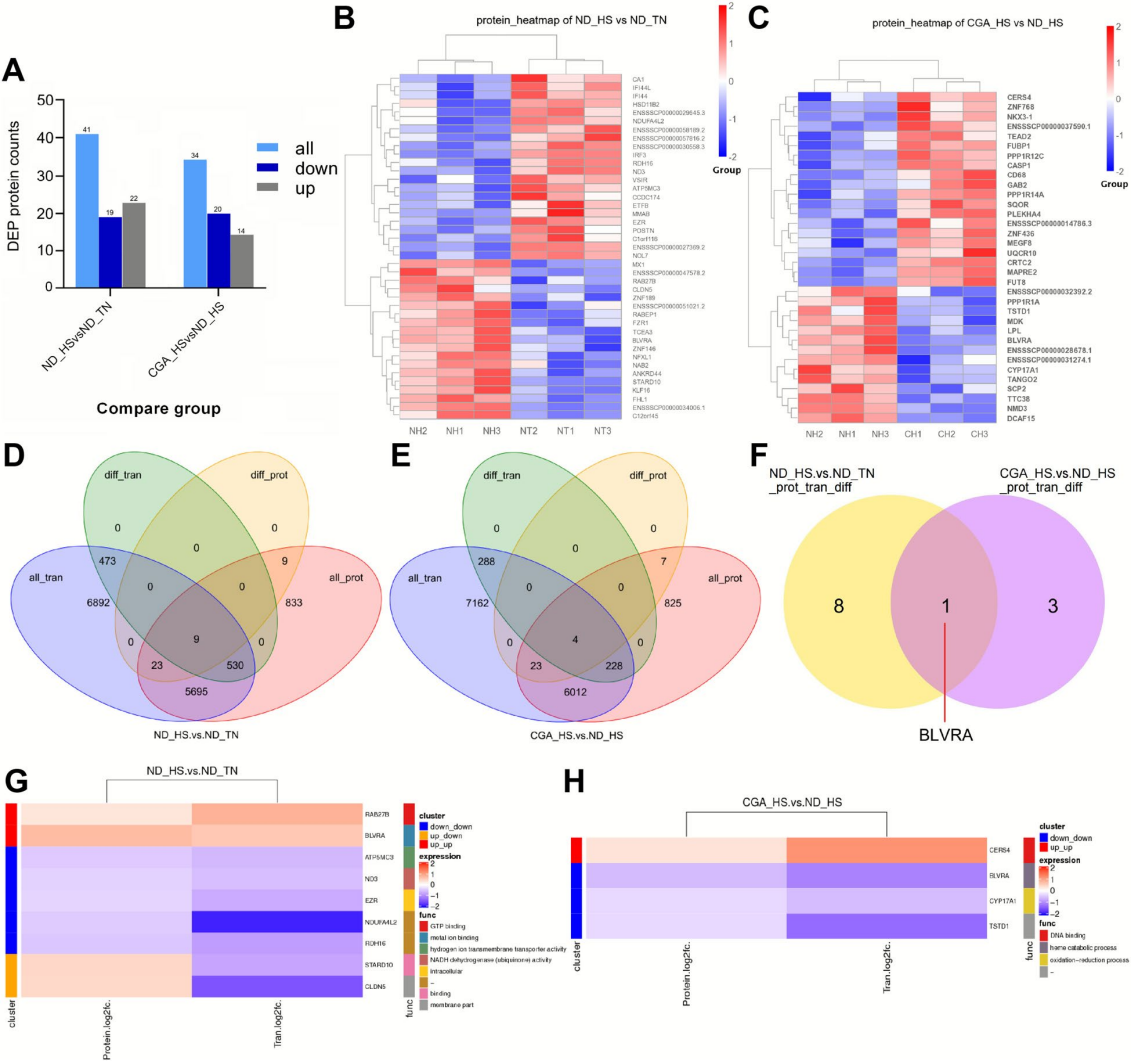
Differential expression profiling of proteins and integrated analysis of transcriptome-proteome in prepubertal boar testes. **A** The number of differential proteins in ND_HS vs. ND_TN and CGA_HS vs. ND_HS groups. **B** The clustering of DAPs in comparable groups of ND_HS vs. ND_TN (NT: ND_TN, NH: ND_HS). **C** The clustering of DAPs in comparable groups of CGA_HS vs. ND_HS (NH: ND_HS, CH: CGA_HS). **D** Venn diagrams of transcriptome and proteome expression regulation of ND_HS vs. ND_TN. **E** Venn diagrams of transcriptome and proteome expression regulation of CGA_HS vs. ND_HS. **F** Venn diagrams of genes that were different and had the same trend of expression in the two comparison groups. **G** GO functional enrichment analysis plots for ND_HS vs. ND_TN. **H** GO functional enrichment analysis plots for CGA_HS vs. ND_HS

### Integration analysis of the transcriptome and proteome of porcine testicular tissues

Proteins are the executors of life function, and we will further analyse testicular tissue through proteomic techniques. As shown in Fig. 5A, 41 differentially expressed proteins (DEPs) were identified between the ND_HS group and ND_TN group, of which 19 were upregulated and 22 were downregulated. A total of 34 DEPs were identified between the CGA_HS and ND_HS groups, of which 20 were upregulated and 14 were downregulated.

We selected one differentially expressed protein in the two comparison groups to verify the sequencing results. The expression levels of CYP17A1 (ENSSSCP 00000011283.2) and EZR (ENSSSCP00000004385.4) DEPs at the protein level were detected by Western blotting. The results are shown in Fig. S2C–E, where the EZR protein validation results are consistent with the sequencing data. Specifically, the expression levels of EZR in the ND_HS group and CGA_HS group were significantly lower than those in the ND_TN group (*P* < 0.01 and *P* < 0.05, respectively), and the expression trend of CYP17A1 was consistent with the sequencing data: the expression level of CYP17A1 in the ND_HS group was higher than that in the CGA_HS group but lower than that in the ND_TN group, indicating that the sequencing results are accurate overall and can be used for further research.

A clustering heatmap was constructed to show the upregulation and downregulation of DEPs in different samples. Clusters of samples are shown longitudinally and clusters of proteins are shown horizontally in each picture. As shown in Fig. 5B and C, the two comparison groups were the ND_HS vs. ND_TN group and the CGA_HS vs. ND_HS group, respectively.

The effects of CGA and HS on testicular tissue are complex biological processes, and the relationships between proteins and mRNAs are not simple linear relationships. To elucidate the molecular mechanism underlying the ability of CGA to alleviate HS-induced testicular injury in pigs, an integrated analysis of the transcriptome and proteomic data was performed for the three groups (ND_TN, ND_HS and CGA_HS). Venn diagrams showing the overlaps of mRNAs in the RNA-seq data and proteins in the proteome between the comparisons revealed the genes influenced by HS and restored by CGA in porcine testes (Fig. 5D and E). Correlation analysis of fold changes between transcriptome and proteome for genes co-identified in both omics datasets is shown in the Fig. S2F and G, with correlation coefficients of 0.085 in ND_HS vs. ND_TN and 0.085 in CGA_HS vs. ND_HS.

The number of related genes in the transcriptome and protein of the ND_HS and ND_TN groups was 6,257. There were 9 genes that were differentially expressed at both the mRNA and protein levels, and their annotations and involved pathways are shown in Fig. 5G. Two genes (RAB27B and BLVRA) were upregulated at both the mRNA and protein levels. These genes were enriched in GTP binding (GO:0005525) and metal ion binding (GO:0046872). Five genes (NDUFA4L2, ND3, RDH16, EZR and ATP5MC3) were downregulated at both the mRNA and protein levels. STARD10 and CLDN5 were upregulated at the mRNA level and downregulated at the protein level.

There were 6,267 related genes in the transcriptome and protein of the CGA_HS group and ND_HS group. There were 4 differentially expressed genes at both the mRNA and protein levels, and 1 gene (CERS4) was upregulated at both the mRNA and protein levels. Three genes (BLVRA, CYP17A1 and TSTD1) were downregulated at both the mRNA and protein levels, among which BLVRA was enriched in heme breakdown (GO:0042167) (Fig. 5H).

Through the combined transcriptome and proteome analysis of the ND_HS and ND_TN comparison groups and the CGA_HS and ND_HS comparison groups, it was found that only BLVRA was simultaneously expressed in the differential mRNA and protein of the two comparison groups (Fig. 5F). BLVRA may play an important role in the ability of CGA to alleviate HS injury to testicular tissue in prepuberty boars.

### CGA alleviated HS injury in Sertoli cells via *BLVRA*

**Fig. 6:**
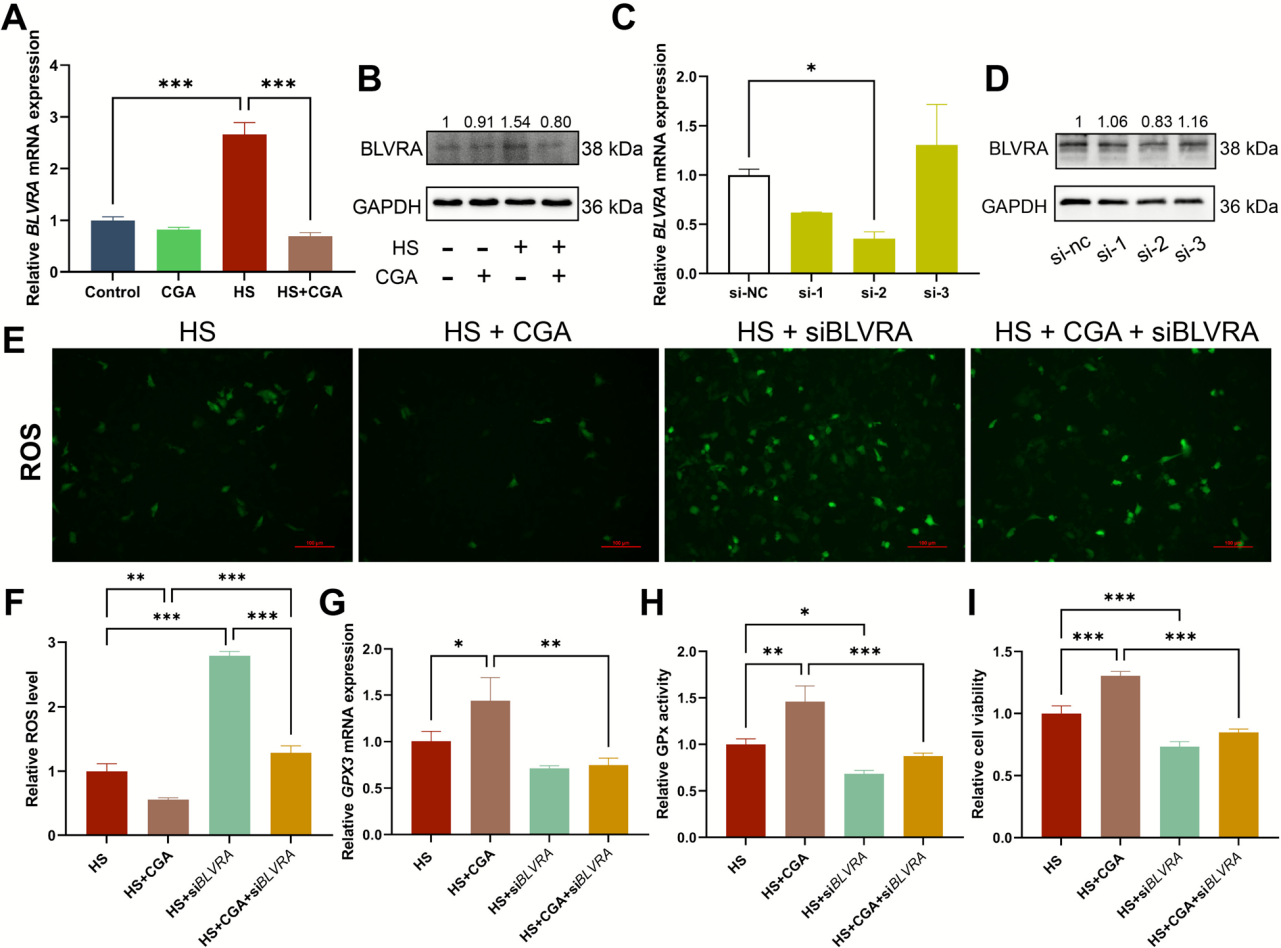
BLVRA is involved in CGA ameliorating HS damage in Sertoli cells. **A** The relative mRNA expression of *BLVRA* in Sertoli cells after CGA and HS treatment in vitro (*n* = 3). **B** The relative protein expression of BLVRA after CGA and HS treatment in vitro. Numbers above the bands represent the relative grayscale values of the corresponding bands. **C** Relative expression level of *BLVRA* mRNA after transfection with interference fragments (*n* = 3). **D** The relative protein expression of BLVRA after transfection with interference fragments. Numbers above the bands represent the relative grayscale values of the corresponding bands. **E** Representative images of ROS in Sertoli cells (20 ×). **F** Relative fluorescence intensity of ROS in cells from different treatment groups (*n* = 3). **G** Relative mRMA expression levels of *GPX3* in cells from different treatment groups (*n* = 3). **H** Relative activity of GPx in cells from different treatment groups (*n* = 3). **I** Relative cell viability of cells from different treatment groups (*n* = 3). Data are expressed as the mean ± SD. ^*^P < 0.05, ^**^P < 0.01, ^***^P < 0.001

Considering the effect of heat stress on the number of Sertoli cells in prepubescent pig testes and the important role of Sertoli cells in testicular development, we chose Sertoli cells to analyse whether CGA could alleviate HS via BLVRA in vitro. Compared with those in the control group, the mRNA (Fig. 6A) and protein (Fig. 6B) expression levels of BLVRA were upregulated after HS (*P* < 0.001), and the addition of CGA alone did not affect the mRNA expression of *BLVRA*. Compared with that in the HS group, the expression of the BLVRA protein in the HS + CGA group was inhibited, whereas the expression of the BLVRA protein decreased (*P* < 0.001). These results indicate that CGA can reduce the increase in BLVRA expression caused by HS, which is consistent with the results of the combined transcriptome and proteome analysis. To assess the role of BLVRA, we used specific siRNAs to interfere with BLVRA expression, and the results demonstrated that si-2 significantly inhibited BLVRA expression compared with si-nc (*P* < 0.05; Fig. 6C and D). Si-2 (hereafter denoted as siBLVRA) was chosen for subsequent experiments because it had the strongest knockdown effect.

ROS fluorescence staining revealed that compared with HS + CGA and HS + siBLVRA, CGA significantly reduced the ROS level in the heat treatment group (HS vs. HS + CGA and HS + siBLVRA vs. HS + CGA + siBLVRA, *P* < 0.001 and *P* < 0.05, respectively). The ROS level was significantly increased in the HS + siBLVRA group (*P* < 0.001), and at the same time, compared with the HS + CGA group, the ROS level was extremely significantly increased in the HS + CGA + siBLVRA group (*P* < 0.001; Fig. 6E and F). RT-qPCR analysis revealed that compared with the HS treatment and the HS + CGA treatment, the CGA treatment resulted in a significant increase in *GPX3* expression (Fig. 6G). However, this effect was not obvious in siBLVRA cells (those in the HS + siBLVRA group compared with those in the HS + CGA + siBLVRA group). GPx activity was next examined to explore whether BLVRA silencing affects glutathione metabolism in Sertoli cells (Fig. 6H). Compared with that in the HS group, the GPx activity in the HS + CGA group was significantly greater (*P* < 0.01), and siBLVRA significantly reduced GPx activity (HS vs. HS + siBLVRA and HS + CGA vs. HS + CGA + siBLVRA, *P* < 0.05 and* P* < 0.001, respectively). Compared with that of the HS group, the cell viability of the HS + CGA group significantly increased (*P* < 0.001), and siBLVRA significantly reduced cell viability (HS vs. HS + siBLVRA and HS + CGA vs. HS + CGA + siBLVRA, *P* < 0.001) (Fig. 6I).

## Discussion

**Fig. 7:**
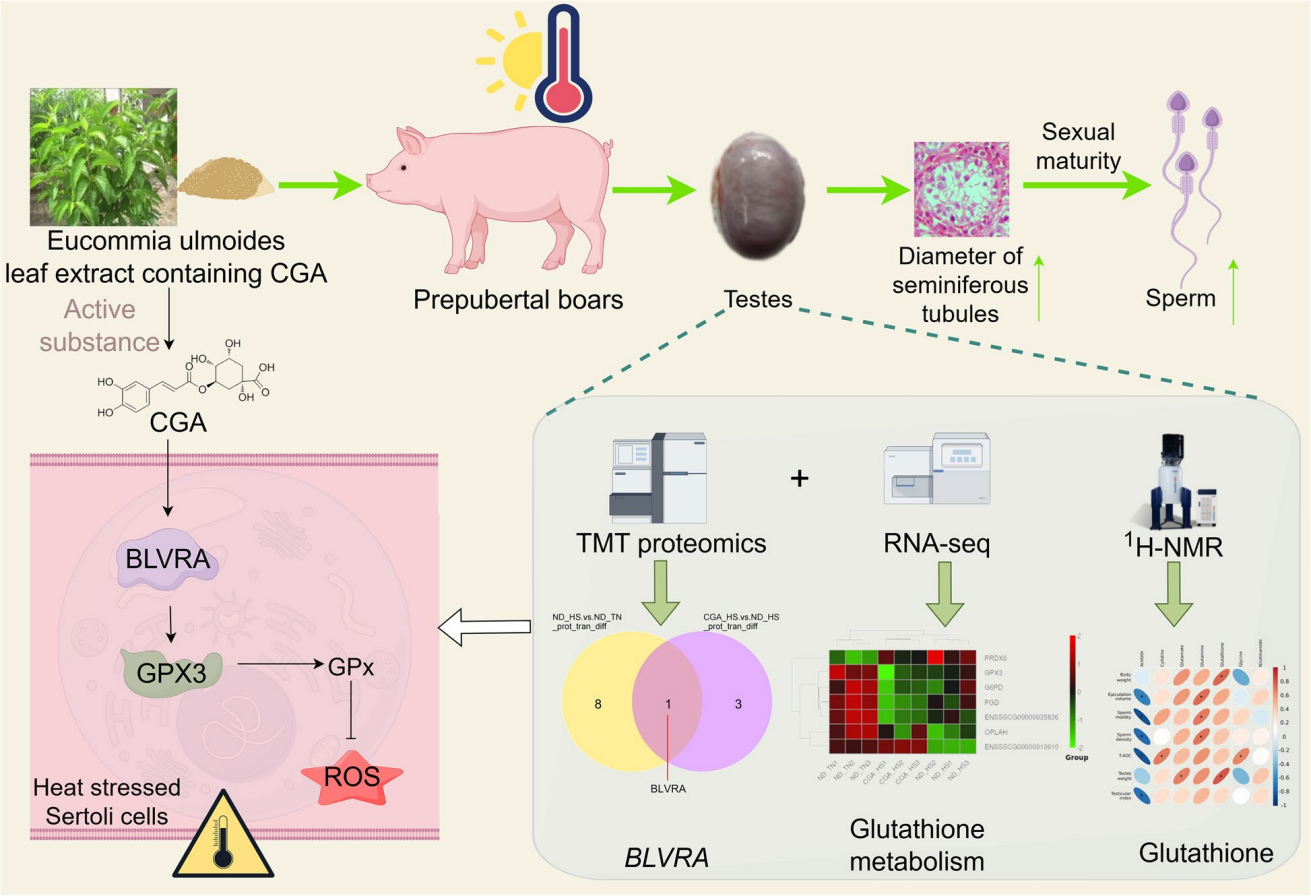
Effects whereby CGA alleviating HS-induced oxidative damage in testes of prepubertal boars. HS during prepubertal can affect the semen quality of male boars after sexual maturity. NMR analysis revealed that the administration of CGA could improve the changes in the levels of glutathione and other metabolites in testicular tissue caused by HS. RNA-seq and TMT technology revealed the changes in gene expression regulated by CGA and HS. In terms of its underlying mechanism, CGA regulates the expression of *GPX3* through BLVRA, affecting GPX activity and alleviating HS injury

Increasing ambient temperature has a serious negative effect on agricultural production and human health [10, 43]. Moreover, this high-temperature climate cannot be alleviated in the short term [44]. This study investigated the testicular tissue damage induced by HS in prepubertal boars and the effect of dietary CGA on heat-stressed testis injury. The results showed that CGA could increase the antioxidant capacity of prepubertal porcine testes and prevent the decrease of glutathione content caused by HS. Through transcriptomic and proteomic analyses, BLVRA emerged as a candidate gene, and our data further suggested that the effect of CGA on HS injury in prepubertal boars may be partially mediated by the potential effect of the BLVRA-GPX3 axis on GPx activity, as determined by transcriptomic and proteomic analyses (Fig. 7).

Currently, several methods are available for constructing a testicular HS model, including whole-body heat exposure treatment, water bath or scrotal local heating treatment, and in vitro HS of testicular tissue [45–47]. In this study, to explore the effects of HS on actual production in prepubertal boars, a whole-body heat exposure method was used to heat-treat prepubertal boars. Through behavioral observations and measurements of body temperature and scrotal temperature, THI, cortisol and HSP70, these results demonstrated that HS-induced anxiety and elevated body temperature in boars, which is consistent with the findings of previous reports [48–51].

The damage caused by HS to the reproductive ability of male animals mainly includes the damage to the structure of the seminiferous tubules and a reduction in semen quality [16]. In reality, male animals at different ages may be affected by HS, and studying the effects of HS on the testicular development of male animals at different stages of sexual maturity is necessary. In 2018, researchers demonstrated that boars exposed to intrauterine HS during maternal gestation (30–60 d) tended to have a decreased testicular area and a significant decrease in total sperm production at the age of 24 weeks after birth, and the decrease in the number of Sertoli cells may be the main reason for this phenomenon [6]. Mete et al. [4] reported that heat treatment caused apoptosis of spermatogenic cells in the seminiferous tubules of prepubertal rats (30 days old), but they did not evaluate whether HS during this period affected spermatogenesis after sexual maturation in animals. Previous studies have shown that semen quality can recover after HS in adult male mammals for some time, such as 5 weeks in boars or 3 months in humans [52, 53]. To the best of our knowledge, no study has investigated HS in prepubescent boars. In addition, compared with mice, boars are more suitable as valuable model animals for studying human biology in terms of the length of testicular development [11]. Therefore, in this study, 10-week-old prepubertal boars were subjected to heat treatment, which is an important stage of testicular development before the sexual maturity of boars [54], and testicular Sertoli cells are in the proliferation stage [36]. Notably, the final number of Sertoli cells determines the number of germ cells and the spermatogenic capacity that a male animal will support as an adult [7]. The results revealed that after HS in prepubertal boars, the total antioxidant capacity of testicular tissue was decreased, and the number of Sertoli cells decreased. The testis weight and testicular index tended to decrease, indicating that HS damaged the structure and function of the prepubertal boar testes and affected the development of the testis and the number of sperm after sexual maturity. These results suggest that the effect of short-term HS on body weight is greater than that on testicular weight and the effect of HS on spermatogenesis is not apparent until sexual maturity. We speculate that the damage to Sertoli cells is related to the decline in semen quality in later stages.

Prevention is more effective than cure in addressing diseases or life-threatening issues. The use of feed additives is a key strategy for preventing HS in production [16]. CGA is a natural active antioxidant, and its anti-HS effects in many species and organs have been gradually revealed [55–57]. CGA can improve the SOD activity of chicken breast meat and alleviate the adverse effects of HS on the growth performance and meat quality of broilers [58]. It also increased serum antioxidant capacity in heat-stressed cows [59]. Consistent with the results of the above studies, CGA improved the antioxidant capacity of prepubertal boars, as indicated by increased T-AOC and GPx activity, indicating that CGA can alleviate HS injury in the testes of prepubertal boars. Next, we explored the mechanism by which CGA alleviates testicular HS injury.

The present study is the first to explain the protective effects of CGA against HS in terms of metabolism, transcription, and translation. Biochemical markers in tissues can reflect the metabolic state of an animal. To investigate how GCA affects the testes of heat-stressed boars at the metabolite level, we first performed ^1^H-NMR metabolomics studies. The results revealed that HS significantly increased the acetate content and decreased the glutamate, glutamine, and glutathione contents. CGA significantly increased the glycine and cytidine contents and decreased the nicotinamide content. Some studies have shown that compared with the offspring of mice fed a normal diet, the F1 generation (offspring) and F2 generation (grandson) of mice fed a high-fat diet (HFD) have a reduced sperm count and changes in the content of metabolites such as acetate. It is speculated that the increase in acetate is related to the self-renewal of germ cells and a decrease in the sperm count [60]. Glutamine is an important energy substrate for Sertoli cells, and depletion of glutamine may impair the ability of Sertoli cells to provide nutritional support to developing germ cells [60, 61]. In addition, glutamine can increase the activity of G6PD, which is a major activator of glutathione-dependent antioxidant defence. Glutamine induces an increase in G6PD activity, increases the level of NADPH, a cofactor necessary for the production of glutathione, and maintains the GSH pool and cellular redox balance, thereby improving sperm density and sperm quality in the seminiferous tubules and epididymal lumen [62]. Other studies revealed that Qrich2 knockout mice had an accumulation of glutamine (Gln) and a decrease in the glutamate concentration in the testis and sperm, dysregulation of amino acid metabolism, and decreased sperm motility. Gln/Glu metabolism maintains the structural stability of microtubules in sperm flagella by regulating the glutamylation level of tubulin, and Qrich2 serves as a possible new Gln sensor to regulate the glutamylation of microtubules and mitochondrial function in mouse sperm [63]. Our results confirmed that HS disrupted the antioxidant balance of testicular tissue and caused testicular damage. Glycine is a nonessential amino acid synthesized by mammalian cells. Studies have shown that dietary glycine supplementation can increase the antioxidant level of broiler chickens under HS [64, 65]. Ruan et al. [66] used high-resolution ^1^H-NMR metabolomics to analyse serum and liver tissue extracts from rats. CGA supplementation can increase glycine levels in rats, and serum glycine and glutathione levels may be useful biomarkers of the biological characteristics of nitrogen metabolism in rats [66]. In this study, the level of glycine in the testicular tissue of heat-stressed boars increased after they were fed CGA, which may be related to this phenomenon. Nicotinamide, a component of coenzymes I and II, is involved in a variety of metabolic pathways (lipid metabolism, oxidation, and anaerobic metabolism). Studies have shown that the relative level of nicotinamide in the testicular Sertoli cells of heat-treated mice decreases after HS treatment [67]. Our results indicate that CGA can ameliorate testicular metabolic imbalance induced by HS in prepubertal boars and that glutamine levels in the prepubertal testis may serve as a potential biomarker for predicting postpubertal sperm density.

Animal phenotypes and metabolomics describe changes in testicular material in prepubescent boars exposed to HS and CGA. Based on transcriptomic data, we preliminarily explored the potential molecular mechanisms underlying these changes and revealed that the glutathione metabolism pathway and ferroptosis pathway may be partially involved in the alleviation of testicular HS by CGA. Previous studies have also shown that glutathione metabolism is affected by HS, which alters the redox balance of the testis [68, 69]. Among them, the expression of *PRDX6*/*GPX3*/*G6PD*/*PGD* and *OPLAH* was altered. Peroxiredoxin 6 (PRDX6) is a member of the peroxiredoxin (Prdxs) family, and plays an important role in maintaining cell homeostasis. Wang et al. [70] reported that the expression of PRDX6 decreased in the epididymis of rats treated with 42 °C for 60 min. The upregulated expression of PRDX6 in the testis after HS in this study may be due to differences caused by different tissues or heat treatment methods, resulting in the regulation of *Prdx6* gene expression by substances such as C/EBPβ or CREB [71]. The *GPX3* gene encodes glutathione peroxidase 3, a selenium protein that catalyzes the oxidation of reduced glutathione (GSH) to oxidized glutathione (GSSG) and participates in glutathione metabolism, maintaining the intracellular redox balance and protecting cells from oxidative stress damage [72]. In addition, in recent years, a number of studies have shown that HS is related to ferroptosis, such as in bovine mammary epithelial cells [73], lung epithelial cells [74], and porcine testicular Sertoli cells [75]. Moreover, some researchers have reported that CGA inhibits ferroptosis in mouse intestinal damage [76] and hypoxic-ischaemic brain damage [77]. Amazingly, the results of this study revealed that CGA may be regulated by ferroptosis to improve HS damage to the testes and that the *TF*, *SLC11A2*, *VDAC2*, *TFRC*, *FTL*, *HMOX1*, *MAP1LC3A* and *STEAP3* genes were involved. Haem oxygenases are rate-limiting enzymes in the metabolism of heme. Their main function is to catabolize haem into carbon monoxide, biliverdin, and Fe^2+^. In recent years, it has been reported that HMOX1 is associated with ferroptosis. For example, Tang et al. [78] reported that sodium iodate induced oxidative stress in retinal pigment epithelial cells through Nrf2-SLC7A11-HMOX1, which regulated the accumulation of Fe^2+^ ions and eventually led to ferroptosis of retinal pigment epithelial cells. This process is the main pathological cause of retinal pigment epithelial degeneration. In addition, other researchers have reported that paeonol can regulate the PI3K/AKT pathway by targeting HMOX1 to reduce oxidized low-density lipoprotein (LDL) induced ferroptosis in human umbilical vein endothelial cells [79]. However, the mechanism of action of these genes in the mitigation of HS injury by CGA remains to be explored. In addition, we found that the expression level of CYP17A1 in the testicular tissues of prepubertal boars was lower in the CGA_HS group than in both the ND_HS and ND_TN groups. As a key enzyme in androgen biosynthesis, CYP17A1 has been shown by Oka et al. [80] to be transcriptionally inhibited by local testicular heat treatment in rats, leading to insufficient testosterone biosynthesis, suggesting that HS may impair Leydig cell function. Unexpectedly, CGA treatment also reduced CYP17A1 expression, which contradicts its effect in male mice [81]. We speculate that this discrepancy may be attributed to species-specific differences or dosage effects, highlighting the need to consider potential side effects at this dosage.

By combined analysis of the transcriptome and proteomic data, *BLVRA* was identified as a candidate mediator through which CGA relieves testicular HS. The BLVRA enzyme can convert biliverdin into bilirubin with antioxidant activity [82]. There is also evidence indicating that it is related to reproductive health. A genome-wide association study (GWAS) on weaned piglet numbers (NW) in Large White pigs revealed *BLVRA* as the sole annotated gene at the significant ALGA0098819 SNP locus, suggesting its involvement in regulating reproductive performance [83]. In support of this, BLVRA is highly expressed in fertile sperm and is potentially correlated with the development of healthy piglet traits [84]. Tissue-specific expression analysis further revealed that BLVRA is enriched in the caput and the corpus epididymis (but has a low expression level in the testis and the tail of the epididymis), indicating that it plays a specific role in the process of epididymal sperm maturation [85]. Mechanistically, BLVRA forms a zinc ion-dependent complex with epididymal sperm binding protein 1 (ELSPBP1) to bind dying sperm, initiating an enzymatic cascade that scavenges ROS released by apoptotic cells [84]. This key function protects viable sperm in the epididymis from oxidative damage, thereby maintaining sperm quality and contributing to overall reproductive capacity. In vitro, our transcriptome sequencing results revealed that CGA inhibited ROS production and increased the expression of *GPX3*, which was enriched in the glutathione metabolic pathway in transcriptome sequencing results, the enzymatic activity of GPx and cell viability when BLVRA was unaffected. When *BLVRA* expression was knocked down, the production of ROS was reduced, but the expression of *GPX3* mRNA, GPx activity and cell viability were lower than those in the HS + CGA group, indicating that siBLVRA increased the sensitivity of cells to HS and that the ability of CGA to alleviate HS was weakened. Therefore, we believe that CGA could alleviate HS through pathways that include but not exclusively through BLVRA.

To date, few studies have investigated the role of dietary CGA in alleviating testicular HS in prepubertal boars. This study is the first to explore the protective effects of dietary CGA in this population and to preliminarily elucidate its molecular mechanisms using multiomics techniques. However, the current research has several limitations that require attention. On the one hand, a single CGA dosage was used, and long-term tracking of boars’ fertility after sexual maturity was not included. Future studies should employ dose-gradient experiments to optimize the concentration, balancing efficacy with potential side effects; however, the duration of HS exposure is short, and its applicability to commercial pig farms under chronic long-term HS conditions remains to be verified. Additionally, the specific regulatory mechanism of BLVRA in the CGA-mediated alleviation of testicular HS is unclear, necessitating further validation of its key role through functional experiments.

## Conclusions

CGA supplementation attenuated HS-induced oxidative stress and partially improved the antioxidant capacity of prepubertal boars, potentially via BLVRA-GPX3 signalling. However, further research including hormonal profiling, full semen evaluation, dose–response trials, and long-term fertility studies, is needed before practical application.

## Supplementary Information

Additional file 1: Fig. S1 Effect of HS and CGA on kinetic parameters of boar sperm. Fig. S2 Representative 600 MHz ^1^H-NMR spectrum and serial numbers of labelled identified metabolites. Fig. S3 Validation of RNA-Seq and proteomics sequencing results and correlation analysis of transcriptome and proteome expression profiles.

Additional file 2: Table S1 Target sequence of *BLVRA* siRNA. Table S2 Primers used for RT-qPCR analysis. Table S3 Effects of CGA and HS on the growth performance of prepubertal boars. Table S4 Average THI during the heat treatment trial. Table S5 Chemical shift assignments of the metabolites observed in the ^1^H NMR spectra of aqueous extract from prepubertal porcine testicles. Table S6 Relative content of the aqueous metabolites in ND_TN, ND_HS and CGA_HS group.

Additional file 3: Table S7 Correlation coefficient and *P*-value for the correlation analysis of seven metabolites and seven traits.

Additional file 4: Table S8 List of DEGs between different groups.

Additional file 5: Table S9 Expression and difference of genes involved in the glutathione metabolic pathway.

Additional file 6: Table S10 Expression and difference of genes involved in the ferroptosis pathway.

Additional file 7: Table S11 Differentially expressed proteins and their corresponding differentially expressed transcripts.

Additional file 8: The full uncropped Western blots images.

## Data Availability

The raw RNA-seq data generated in this study have been deposited in the NCBI Sequence Read Archive (SRA, https://www.ncbi.nlm.nih.gov/sra) under the accession number PRJNA1246667. The mass spectrometry proteomics data have been deposited to the ProteomeXchange Consortium via the iProX partner repository (https://proteomecentral.proteomexchange.org) under the accession number PXD062793. The ^1^H-NMR spectroscopy data have been deposited in the OMIX, China National Center for Bioinformation/Beijing Institute of Genomics, Chinese Academy of Sciences (https://ngdc.cncb.ac.cn/omix) under the accession number OMIX012729. Apart from the datasets included in the article/supplementary materials, all other data generated in this study are available from the corresponding author upon request.

## Abbreviations

^1^H-NMR: One-dimensional nuclear magnetic resonance hydrogen spectrum
CGA: Chlorogenic acid
GO: Gene Ontology
HS: Heat stress
KEGG: Kyoto Encyclopedia of Genes and Genomes
ND: Normal diet
ROS: Reactive oxygen species
T-AOC: Total antioxidant capacity
TMT: Tandem mass tag
TN: Thermoneutral

## References

[1] Matthews HD, Wynes S. Current global efforts are insufficient to limit warming to 1.5 degrees C. Science. 2022;376(6600):1404–9. 10.1126/science.abo3378. 35737785 10.1126/science.abo3378

[2] Kim B, Park K, Rhee K. Heat stress response of male germ cells. Cell Mol Life Sci. 2013;70(15):2623–36. 10.1007/s00018-012-1165-4. 23007846 10.1007/s00018-012-1165-4 PMC11113252

[3] Hedia MG, El-Belely MS, Ismail ST, Abo El-Maaty AM. Seasonal variation in testicular blood flow dynamics and their relation to systemic and testicular oxidant/antioxidant biomarkers and androgens in rams. Reprod Domest Anim. 2020;55(7):861–9. 10.1111/rda.13696. 32374490 10.1111/rda.13696

[4] Mete F, Kilic E, Somay A, Yilmaz B. Effects of heat stress on endocrine functions & behaviour in the pre-pubertal rat. Indian J Med Res. 2012;135(2):233–9. 22446867 PMC3336856

[5] Sabes-Alsina M, Lundeheim N, Johannisson A, Lopez-Bejar M, Morrell JM. Relationships between climate and sperm quality in dairy bull semen: a retrospective analysis. J Dairy Sci. 2019;102(6):5623–33. 10.3168/jds.2018-15837. 30904295 10.3168/jds.2018-15837

[6] Lugar DW, Proctor JA, Safranski TJ, Lucy MC, Stewart KR. In utero heat stress causes reduced testicular area at puberty, reduced total sperm production, and increased sperm abnormalities in boars. Anim Reprod Sci. 2018;192:126–35. 10.1016/j.anireprosci.2018.02.022. 29567201 10.1016/j.anireprosci.2018.02.022

[7] Flowers WL. Factors affecting the production of quality ejaculates from boars. Anim Reprod Sci. 2022;246:106840. 10.1016/j.anireprosci.2021.106840. 34518030 10.1016/j.anireprosci.2021.106840

[8] Shakeel M, Yoon M. Changes in characteristics of spermatogonial stem cells in response to heat stress in stallions. Theriogenology. 2024;224:74–81. 10.1016/j.theriogenology.2024.05.007. 38759607 10.1016/j.theriogenology.2024.05.007

[9] Raoofi A, Omraninava M, Javan R, Maghsodi D, Rustamzadeh A, Nasiry D, et al. Protective effects of epigallocatechin gallate in the mice induced by chronic scrotal hyperthermia. Tissue Cell. 2023;84:102165. 10.1016/j.tice.2023.102165. 37480630 10.1016/j.tice.2023.102165

[10] Ko SH. Effects of heat stress-induced sex hormone dysregulation on reproduction and growth in male adolescents and beneficial foods. Nutrients. 2024;16(17):3032. 10.3390/nu16173032. 10.3390/nu16173032 PMC11397449 39275346

[11] Voigt AL, Dardari R, Lara NLM, He T, Steele H, Dufour A, et al. Multiomics approach to profiling Sertoli cell maturation during development of the spermatogonial stem cell niche. Mol Hum Reprod. 2023;29(3):gaad004. 10.1093/molehr/gaad004. 10.1093/molehr/gaad004 PMC9976880 36688722

[12] Zheng Y, Gao Q, Li T, Liu R, Cheng Z, Guo M, et al. Sertoli cell and spermatogonial development in pigs. J Anim Sci Biotechnol. 2022;13:45. 10.1186/s40104-022-00687-2. 35399096 10.1186/s40104-022-00687-2 PMC8996595

[13] Hu H, Dai S, Li J, Wen A, Bai X. Glutamine improves heat stress-induced oxidative damage in the broiler thigh muscle by activating the nuclear factor erythroid 2-related 2/Kelch-like ECH-associated protein 1 signaling pathway. Poult Sci. 2020;99(3):1454–61. 10.1016/j.psj.2019.11.001. 32115031 10.1016/j.psj.2019.11.001 PMC7587763

[14] Zhang P, Zheng Y, Lv Y, Li F, Su L, Qin Y, et al. Melatonin protects the mouse testis against heat-induced damage. Mol Hum Reprod. 2020;26(2):65–79. 10.1093/molehr/gaaa002. 31943111 10.1093/molehr/gaaa002

[15] Hu Y, Luo NJ, Gan L, Xue HY, Luo KY, Zhang JJ, et al. Heat stress upregulates arachidonic acid to trigger autophagy in Sertoli cells via dysfunctional mitochondrial respiratory chain function. J Transl Med. 2024;22(1):501. 10.1186/s12967-024-05182-y. 38797842 10.1186/s12967-024-05182-y PMC11129461

[16] Shahat AM, Rizzoto G, Kastelic JP. Amelioration of heat stress-induced damage to testes and sperm quality. Theriogenology. 2020;158:84–96. 10.1016/j.theriogenology.2020.08.034. 32947064 10.1016/j.theriogenology.2020.08.034

[17] Wang S, Li Y, Meng X, Chen S, Huang D, Xia Y, et al. Antioxidant activities of chlorogenic acid derivatives with different acyl donor chain lengths and their stabilities during in vitro simulated gastrointestinal digestion. Food Chem. 2021;357:129904. 10.1016/j.foodchem.2021.129904. 33915469 10.1016/j.foodchem.2021.129904

[18] Zhang S, Sun B, Wang D, Liu Y, Li J, Qi J, et al. Chlorogenic acid ameliorates damage induced by Fluorene-9-Bisphenol in porcine Sertoli cells. Front Pharmacol. 2021;12:678772. 10.3389/fphar.2021.678772. 34177588 10.3389/fphar.2021.678772 PMC8219976

[19] Hu R, Yang X, Wang L, Su D, He Z, Li J, et al. Gut microbiota dysbiosis and oxidative damage in high-fat diet-induced impairment of spermatogenesis: role of protocatechuic acid intervention. Food Frontiers. 2024;5(6):2566–78. 10.1002/fft2.484.

[20] Hu R, Yang X, Gong J, Lv J, Yuan X, Shi M, et al. Patterns of alteration in boar semen quality from 9 to 37 months old and improvement by protocatechuic acid. J Anim Sci Biotechnol. 2024;15:78. 10.1186/s40104-024-01031-6. 38755656 10.1186/s40104-024-01031-6 PMC11100174

[21] Zhang SX, Wang DL, Qi JJ, Yang YW, Sun H, Sun BX, et al. Chlorogenic acid ameliorates the heat stress-induced impairment of porcine Sertoli cells by suppressing oxidative stress and apoptosis. Theriogenology. 2024;214:148–56. 10.1016/j.theriogenology.2023.10.018. 37875054 10.1016/j.theriogenology.2023.10.018

[22] Kim H, Pan JH, Kim SH, Lee JH, Park JW. Chlorogenic acid ameliorates alcohol-induced liver injuries through scavenging reactive oxygen species. Biochimie. 2018;150:131–8. 10.1016/j.biochi.2018.05.008. 29787793 10.1016/j.biochi.2018.05.008

[23] Zhou Y, Zhou L, Ruan Z, Mi S, Jiang M, Li X, et al. Chlorogenic acid ameliorates intestinal mitochondrial injury by increasing antioxidant effects and activity of respiratory complexes. Biosci Biotechnol Biochem. 2016;80(5):962–71. 10.1080/09168451.2015.1127130. 26824685 10.1080/09168451.2015.1127130

[24] Li H, Zhao J, Deng W, Li K, Liu H. Effects of chlorogenic acid-enriched extract from *Eucommia ulmoides* Oliver leaf on growth performance and quality and oxidative status of meat in finishing pigs fed diets containing fresh or oxidized corn oil. J Anim Physiol Anim Nutr (Berl). 2020;104(4):1116–25. 10.1111/jpn.13267. 31802552 10.1111/jpn.13267

[25] Chen J, Li Y, Yu B, Chen D, Mao X, Zheng P, et al. Dietary chlorogenic acid improves growth performance of weaned pigs through maintaining antioxidant capacity and intestinal digestion and absorption function. J Anim Sci. 2018;96(3):1108–18. 10.1093/jas/skx078. 29562339 10.1093/jas/skx078 PMC6093540

[26] Zhang Y, Qu H, Pan H, Xiang D, Choi S, Liang S. Effects of supplementation with chlorogenic acid-rich extract from *Eucommia ulmoides* oliver during peri-implantation on the reproductive performance and gut microbiota of sows. Vet Sci. 2025;12(9):857. 10.3390/vetsci12090857. 10.3390/vetsci12090857 PMC12474264 41012782

[27] Consolo NRB, Silva JD, Buarque VLM, Higuera-Padilla A, Barbosa L, Zawadzki A, et al. Selection for growth and precocity alters muscle metabolism in Nellore cattle. Metabolites. 2020;10(2):58. 10.3390/metabo10020058. 10.3390/metabo10020058 PMC7073857 32041181

[28] Chen H, Buhler K, Zhu Y, Nie X, Liu W. Proteomics analysis reveals the effect of 1α,25(OH)_2_VD_3_-glycosides on development of early testes in piglets. Sci Rep. 2021;11:11341. 10.1038/s41598-021-90676-8. 10.1038/s41598-021-90676-8 PMC8167176 34059707

[29] Yang CX, Chen L, Yang YW, Mou Q, Du ZQ. Acute heat stress reduces viability but increases lactate secretion of porcine immature Sertoli cells through transcriptome reprogramming. Theriogenology. 2021;173:183–92. 10.1016/j.theriogenology.2021.06.024. 34392171 10.1016/j.theriogenology.2021.06.024

[30] Nguyen N, Jennen D, Kleinjans J. Omics technologies to understand drug toxicity mechanisms. Drug Discov Today. 2022;27(11):103348. 10.1016/j.drudis.2022.103348. 36089240 10.1016/j.drudis.2022.103348

[31] Chen H, Zhang L, Liu M, Li Y, Chi Y. Multi-omics research on angina pectoris: A novel perspective. Aging Dis. 2024;16(6):3381–99. 10.14336/AD.2024.1298. 39751862 10.14336/AD.2024.1298 PMC12539525

[32] Long JA. The “omics” revolution: use of genomic, transcriptomic, proteomic and metabolomic tools to predict male reproductive traits that impact fertility in livestock and poultry. Anim Reprod Sci. 2020;220:106354. 10.1016/j.anireprosci.2020.106354. 32482486 10.1016/j.anireprosci.2020.106354

[33] Fan X, Xi H, Zhang Z, Liang Y, Li Q, He J. Germ cell apoptosis and expression of Bcl-2 and Bax in porcine testis under normal and heat stress conditions. Acta Histochem. 2017;119(3):198–204. 10.1016/j.acthis.2016.09.003. 28279507 10.1016/j.acthis.2016.09.003

[34] Mader TL, Johnson LJ, Gaughan JB. A comprehensive index for assessing environmental stress in animals. J Anim Sci. 2010;88(6):2153–65. 10.2527/jas.2009-2586. 20118427 10.2527/jas.2009-2586

[35] Ding H, Luo Y, Liu M, Huang J, Xu D. Histological and transcriptome analyses of testes from Duroc and Meishan boars. Sci Rep. 2016;6:20758. 10.1038/srep20758. 26865000 10.1038/srep20758 PMC4749976

[36] Franca LR, Silva VA Jr, Chiarini-Garcia H, Garcia SK, Debeljuk L. Cell proliferation and hormonal changes during postnatal development of the testis in the pig. Biol Reprod. 2000;63(6):1629–36. 10.1095/biolreprod63.6.1629. 11090429 10.1095/biolreprod63.6.1629

[37] Yu J, Xiang JY, Xiang H, Xie Q. Cecal butyrate (not propionate) was connected with metabolism-related chemicals of mice, based on the different effects of the two *Inonotus obliquus* extracts on obesity and their mechanisms. ACS Omega. 2020;5(27):16690–700. 10.1021/acsomega.0c01566. 32685836 10.1021/acsomega.0c01566 PMC7364710

[38] Ebrahimi F, Ibrahim B, Teh CH, Murugaiyah V, Chan KL. Urinary NMR-based metabolomic analysis of rats possessing variable sperm count following orally administered *Eurycoma longifolia* extracts of different quassinoid levels. J Ethnopharmacol. 2016;182:80–9. 10.1016/j.jep.2016.02.015. 26899442 10.1016/j.jep.2016.02.015

[39] Griffin JL, Troke J, Walker LA, Shore RF, Lindon JC, Nicholson JK. The biochemical profile of rat testicular tissue as measured by magic angle spinning ^1^H NMR spectroscopy. FEBS Lett. 2000;486(3):225–9. 10.1016/s0014-5793(00)02307-3. 11119708 10.1016/s0014-5793(00)02307-3

[40] Haritwal T, Maan K, Rana P, Parvez S, Singh AK, Khushu S, et al. Trichostatin A, an epigenetic modifier, mitigates radiation-induced androphysiological anomalies and metabolite changes in mice as evident from NMR-based metabolomics. Int J Radiat Biol. 2019;95(4):443–51. 10.1080/09553002.2018.1524989. 30307353 10.1080/09553002.2018.1524989

[41] Islam R, Melvin SD, Yu RMK, O'Connor WA, Tran TKA, Andrew-Priestley M, et al. Exposure to estrogenic mixtures results in tissue-specific alterations to the metabolome of oysters. Aquat Toxicol. 2021;231:105722. 10.1016/j.aquatox.2020.105722. 10.1016/j.aquatox.2020.105722 33360311

[42] Straadt IK, Young JF, Bross P, Gregersen N, Oksbjerg N, Theil PK, et al. NMR-based metabonomic investigation of heat stress in myotubes reveals a time-dependent change in the metabolites. J Agric Food Chem. 2010;58(10):6376–86. 10.1021/jf904197u. 20429597 10.1021/jf904197u

[43] Gujar G, Tiwari M, Yadav N, Monika D. Heat stress adaptation in cows - Physiological responses and underlying molecular mechanisms. J Therm Biol. 2023;118:103740. 10.1016/j.jtherbio.2023.103740. 37976864 10.1016/j.jtherbio.2023.103740

[44] Zhou J, Zhao JH, Li YH, Bai YY, Wu Y, Xiang B, et al. The hottest center: characteristics of high temperatures in midsummer of 2022 in Chongqing and its comparison with 2006. Theor Appl Climatol. 2024;155(1):151–62. 10.1007/s00704-023-04609-8.

[45] Li Y, Li Z, Cao Y, Zhou X, Li C. Chronic excessive Zn intake increases the testicular sensitivity to high ambient temperature in Bama miniature pigs. Environ Pollut. 2020;257:113629. 10.1016/j.envpol.2019.113629. 31806468 10.1016/j.envpol.2019.113629

[46] Shen H, Fan X, Zhang Z, Xi H, Ji R, Liu Y, et al. Effects of elevated ambient temperature and local testicular heating on the expressions of heat shock protein 70 and androgen receptor in boar testes. Acta Histochem. 2019;121(3):297–302. 10.1016/j.acthis.2019.01.009. 30723046 10.1016/j.acthis.2019.01.009

[47] Wang K, Li Z, Li Y, Li X, Suo Y, Li C. Impacts of elevated temperature on morphology, oxidative stress levels, and testosterone synthesis in ex vivo cultured porcine testicular tissue. Theriogenology. 2023;212:181–8. 10.1016/j.theriogenology.2023.09.015. 37742481 10.1016/j.theriogenology.2023.09.015

[48] Gong S, Miao YL, Jiao GZ, Sun MJ, Li H, Lin J, et al. Dynamics and correlation of serum cortisol and corticosterone under different physiological or stressful conditions in mice. PLoS ONE. 2015;10(2):e0117503. 10.1371/journal.pone.0117503. 25699675 10.1371/journal.pone.0117503 PMC4336318

[49] Michel V, Peinnequin A, Alonso A, Buguet A, Cespuglio R, Canini F. Decreased heat tolerance is associated with hypothalamo-pituitary-adrenocortical axis impairment. Neuroscience. 2007;147(2):522–31. 10.1016/j.neuroscience.2007.04.035. 17531395 10.1016/j.neuroscience.2007.04.035

[50] Bouchama A, Knochel JP. Heat stroke. N Engl J Med. 2002;346(25):1978–88. 10.1056/NEJMra011089. 12075060 10.1056/NEJMra011089

[51] Veilleux JC, Zielinski MJ, Moyen NE, Tucker MA, Dougherty EK, Ganio MS. The effect of passive heat stress on distress andself-control in male smokers and non-smokers. J Gen Psychol. 2018;145(4):342–61. 10.1080/00221309.2018.1494127. 30358519 10.1080/00221309.2018.1494127

[52] Garolla A, Torino M, Sartini B, Cosci I, Patassini C, Carraro U, et al. Seminal and molecular evidence that sauna exposure affects human spermatogenesis. Hum Reprod. 2013;28(4):877–85. 10.1093/humrep/det020. 23411620 10.1093/humrep/det020

[53] Wettemann RP, Bazer FW. Influence of environmental temperature on prolificacy of pigs. J Reprod Fertil Suppl. 1985;33:199–208. 3910825

[54] Lervik S, Kristoffersen AB, Conley LN, Oskam IC, Hedegaard J, Ropstad E, et al. Gene expression during testis development in Duroc boars. Animal. 2015;9(11):1832–42. 10.1017/S1751731115000907. 26016904 10.1017/S1751731115000907

[55] Chen F, Zhang H, Zhao N, Yang X, Du E, Huang S, et al. Effect of chlorogenic acid on intestinal inflammation, antioxidant status, and microbial community of young hens challenged with acute heat stress. Anim Sci J. 2021;92(1):e13619. 10.1111/asj.13619. 34409681 10.1111/asj.13619

[56] Ji R, Chen J, Xu J, Zhang L, Liu L, Li F. Protective effect of chlorogenic acid on liver injury in heat-stressed meat rabbits. J Anim Physiol Anim Nutr (Berl). 2024;108(5):1203–13. 10.1111/jpn.13966. 38628061 10.1111/jpn.13966

[57] Nguyen TV, Do LTK, Somfai T, Otoi T, Taniguchi M, Kikuchi K. Presence of chlorogenic acid during in vitro maturation protects porcine oocytes from the negative effects of heat stress. Anim Sci J. 2019;90(12):1530–6. 10.1111/asj.13302. 31663235 10.1111/asj.13302

[58] Zhao JS, Deng W, Liu HW. Effects of chlorogenic acid-enriched extract from *Eucommia ulmoides* leaf on performance, meat quality, oxidative stability, and fatty acid profile of meat in heat-stressed broilers. Poult Sci. 2019;98(7):3040–9. 10.3382/ps/pez081. 30839075 10.3382/ps/pez081

[59] Ma F, Liu J, Li S, Sun P. Effects of *Lonicera japonica* extract with different contents of chlorogenic acid on lactation performance, serum parameters, and rumen fermentation in heat-stressed Holstein high-yielding dairy cows. Animals (Basel). 2024;14(8):1252. 10.3390/ani14081252. 10.3390/ani14081252 PMC11047513 38672400

[60] Crisostomo L, Jarak I, Rato LP, Raposo JF, Batterham RL, Oliveira PF, et al. Inheritable testicular metabolic memory of high-fat diet causes transgenerational sperm defects in mice. Sci Rep. 2021;11:9444. 10.1038/s41598-021-88981-3. 33941835 10.1038/s41598-021-88981-3 PMC8093209

[61] Oliveira PF, Martins AD, Moreira AC, Cheng CY, Alves MG. The Warburg effect revisited–lesson from the Sertoli cell. Med Res Rev. 2015;35(1):126–51. 10.1002/med.21325. 25043918 10.1002/med.21325 PMC4845724

[62] Hamed MA, Akhigbe TM, Akhigbe RE, Aremu AO, Oyedokun PA, Gbadamosi JA, et al. Glutamine restores testicular glutathione-dependent antioxidant defense and upregulates NO/cGMP signaling in sleep deprivation-induced reproductive dysfunction in rats. Biomed Pharmacother. 2022;148:112765. 10.1016/j.biopha.2022.112765. 35247715 10.1016/j.biopha.2022.112765

[63] Zhang G, Guo J, Yang H, Li Q, Ye F, Song Y, et al. Metabolic profiling identifies Qrich2 as a novel glutamine sensor that regulates microtubule glutamylation and mitochondrial function in mouse sperm. Cell Mol Life Sci. 2024;81(1):170. 10.1007/s00018-024-05177-4. 38597976 10.1007/s00018-024-05177-4 PMC11006759

[64] Awad EA, Idrus Z, Soleimani Farjam A, Bello AU, Jahromi MF. Growth performance, duodenal morphology and the caecal microbial population in female broiler chickens fed glycine-fortified low protein diets under heat stress conditions. Br Poult Sci. 2018;59(3):340–8. 10.1080/00071668.2018.1440377. 29433333 10.1080/00071668.2018.1440377

[65] Deng C, Zheng J, Zhou H, You J, Li G. Dietary glycine supplementation prevents heat stress-induced impairment of antioxidant status and intestinal barrier function in broilers. Poult Sci. 2023;102(3):102408. 10.1016/j.psj.2022.102408. 36584416 10.1016/j.psj.2022.102408 PMC9827071

[66] Ruan Z, Yang Y, Zhou Y, Wen Y, Ding S, Liu G, et al. Metabolomic analysis of amino acid and energy metabolism in rats supplemented with chlorogenic acid. Amino Acids. 2014;46(9):2219–29. 10.1007/s00726-014-1762-7. 24927697 10.1007/s00726-014-1762-7 PMC5013734

[67] Xu B, Chen M, Ji X, Yao M, Mao Z, Zhou K, et al. Metabolomic profiles reveal key metabolic changes in heat stress-treated mouse Sertoli cells. Toxicol In Vitro. 2015;29(7):1745–52. 10.1016/j.tiv.2015.07.009. 26165742 10.1016/j.tiv.2015.07.009

[68] Cai XQ, Yang H, Liang BQ, Deng CC, Xue HY, Zhang JJ, et al. Glutamate rescues heat stress-induced apoptosis of Sertoli cells by enhancing the activity of antioxidant enzymes and activating the Trx1-Akt pathway in vitro. Theriogenology. 2024;223:1–10. 10.1016/j.theriogenology.2024.04.005. 38642435 10.1016/j.theriogenology.2024.04.005

[69] Chen N, Huang Z, Lu C, Shen Y, Luo X, Ke C, et al. Different transcriptomic responses to thermal stress in heat-tolerant and heat-sensitive Pacific abalones indicated by cardiac performance. Front Physiol. 2018;9:1895. 10.3389/fphys.2018.01895. 30687115 10.3389/fphys.2018.01895 PMC6334008

[70] Wang X, Liu F, Gao X, Liu X, Kong X, Wang H, et al. Comparative proteomic analysis of heat stress proteins associated with rat sperm maturation. Mol Med Rep. 2016;13(4):3547–52. 10.3892/mmr.2016.4958. 26936680 10.3892/mmr.2016.4958

[71] Wu X, Ji P, Zhang L, Bu G, Gu H, Wang X, et al. The expression of porcine *Prdx6* gene is up-regulated by C/EBPβ and CREB. PLoS ONE. 2015;10(12):e0144851. 10.1371/journal.pone.0144851. 26659441 10.1371/journal.pone.0144851 PMC4699452

[72] Moulder R, Hirvonen MK, Valikangas T, Suomi T, Overbergh L, Peakman M, et al. Targeted serum proteomics of longitudinal samples from newly diagnosed youth with type 1 diabetes affirms markers of disease. Diabetologia. 2025. 10.1007/s00125-025-06394-7. 40019499 10.1007/s00125-025-06394-7 PMC12069125

[73] Xu J, Wang XL, Zeng HF, Han ZY. Methionine alleviates heat stress-induced ferroptosis in bovine mammary epithelial cells through the Nrf2 pathway. Ecotoxicol Environ Saf. 2023;256:114889. 10.1016/j.ecoenv.2023.114889. 37079940 10.1016/j.ecoenv.2023.114889

[74] Chen H, Lin X, Yi X, Liu X, Yu R, Fan W, et al. SIRT1-mediated p53 deacetylation inhibits ferroptosis and alleviates heat stress-induced lung epithelial cells injury. Int J Hyperthermia. 2022;39(1):977–86. 10.1080/02656736.2022.2094476. 35853732 10.1080/02656736.2022.2094476

[75] Yang H, Cai X, Qiu M, Deng C, Xue H, Zhang J, et al. Heat stress induces ferroptosis of porcine Sertoli cells by enhancing CYP2C9-Ras- JNK axis. Theriogenology. 2024;215:281–9. 10.1016/j.theriogenology.2023.11.027. 38103405 10.1016/j.theriogenology.2023.11.027

[76] Zhao Y, Wang C, Yang T, Wang H, Zhao S, Sun N, et al. Chlorogenic acid alleviates chronic stress-induced duodenal ferroptosis via the inhibition of the IL-6/JAK2/STAT3 signaling pathway in rats. J Agric Food Chem. 2022;70(14):4353–61. 10.1021/acs.jafc.2c01196. 35380825 10.1021/acs.jafc.2c01196

[77] Li LY, Wang Q, Deng L, Lin Z, Lin JJ, Wang XY, et al. Chlorogenic acid alleviates hypoxic-ischemic brain injury in neonatal mice. Neural Regen Res. 2023;18(3):568–76. 10.4103/1673-5374.350203. 36018179 10.4103/1673-5374.350203 PMC9727453

[78] Tang Z, Ju Y, Dai X, Ni N, Liu Y, Zhang D, et al. HO-1-mediated ferroptosis as a target for protection against retinal pigment epithelium degeneration. Redox Biol. 2021;43:101971. 10.1016/j.redox.2021.101971. 33895485 10.1016/j.redox.2021.101971 PMC8099560

[79] Guo W, Yang H, He W. Paeonol alleviates ox-LDL-induced endothelial cell injury by targeting the heme oxygenase-1/phosphoinositide 3-kinase/protein kinase B pathway. Naunyn Schmiedebergs Arch Pharmacol. 2025;398(1):591–600. 10.1007/s00210-024-03307-0. 39037459 10.1007/s00210-024-03307-0

[80] Oka S, Shiraishi K, Fujimoto M, Katiyar A, Takii R, Nakai A, et al. Role of heat shock factor 1 in conserving cholesterol transportation in Leydig cell steroidogenesis via steroidogenic acute regulatory protein. Endocrinology. 2017;158(8):2648–58. 10.1210/en.2017-00132. 28575284 10.1210/en.2017-00132

[81] Zheng HX, Xu YM, Fan SC, Qi SS, Jia FF, Wu W, et al. Potential protective role of chlorogenic acid against cyclophosphamide-induced reproductive damage in male mice. Toxicol Res. 2024;13(5):tfae176. 10.1093/toxres/tfae176. 10.1093/toxres/tfae176 PMC11519035 39478806

[82] Kim SJ, Shin MJ, Kim DW, Yeo HJ, Yeo EJ, Choi YJ, et al. Tat-biliverdin reductase A exerts a protective role in oxidative stress-induced hippocampal neuronal cell damage by regulating the apoptosis and MAPK signaling. Int J Mol Sci. 2020;21(8):2672. 10.3390/ijms21082672. 10.3390/ijms21082672 PMC7215548 32290442

[83] Zhang H, Bao S, Zhao X, Bai Y, Lv Y, Gao P, et al. Genome-Wide Association Study and Phenotype Prediction of Reproductive Traits in Large White Pigs. Animals (Basel). 2024;14(23):3348. 10.3390/ani14233348. 10.3390/ani14233348 PMC11639466 39682314

[84] Sullivan R. Epididymosomes: a heterogeneous population of microvesicles with multiple functions in sperm maturation and storage. Asian J Androl. 2015;17(5):726–9. 10.4103/1008-682X.155255. 26112475 10.4103/1008-682X.155255 PMC4577580

[85] D’Amours O, Frenette G, Caron P, Belleannee C, Guillemette C, Sullivan R. Evidences of biological functions of biliverdin reductase A in the bovine epididymis. J Cell Physiol. 2016;231(5):1077–89. 10.1002/jcp.25200. 26395865 10.1002/jcp.25200

