# Supplementary material for: Multiomics analysis reveals that chlorogenic acid alleviates heat stress-induced oxidative damage in prepubertal boar testes via the BLVRA-GPX3 pathway: in vivo and in vitro evidence
Source: J Anim Sci Biotechnol. 2026 Jan 13;17:7. doi: 10.1186/s40104-025-01336-0 (PMC12798073; doi:10.1186/s40104-025-01336-0)
Supplement: Supplementary file 1 — Additional file 1: Fig. S1 Effect of HS and CGA on kinetic parameters of boar sperm. Fig. S2 Representative 600 MHz 1H-NMR spectrum and serial numbers of labelled identified metabolites. Fig. S3 Validation of RNA-Seq and proteomics sequencing results and correlation analysis of transcriptome and proteome expression profiles. [file 40104_2025_1336_MOESM1_ESM.docx]

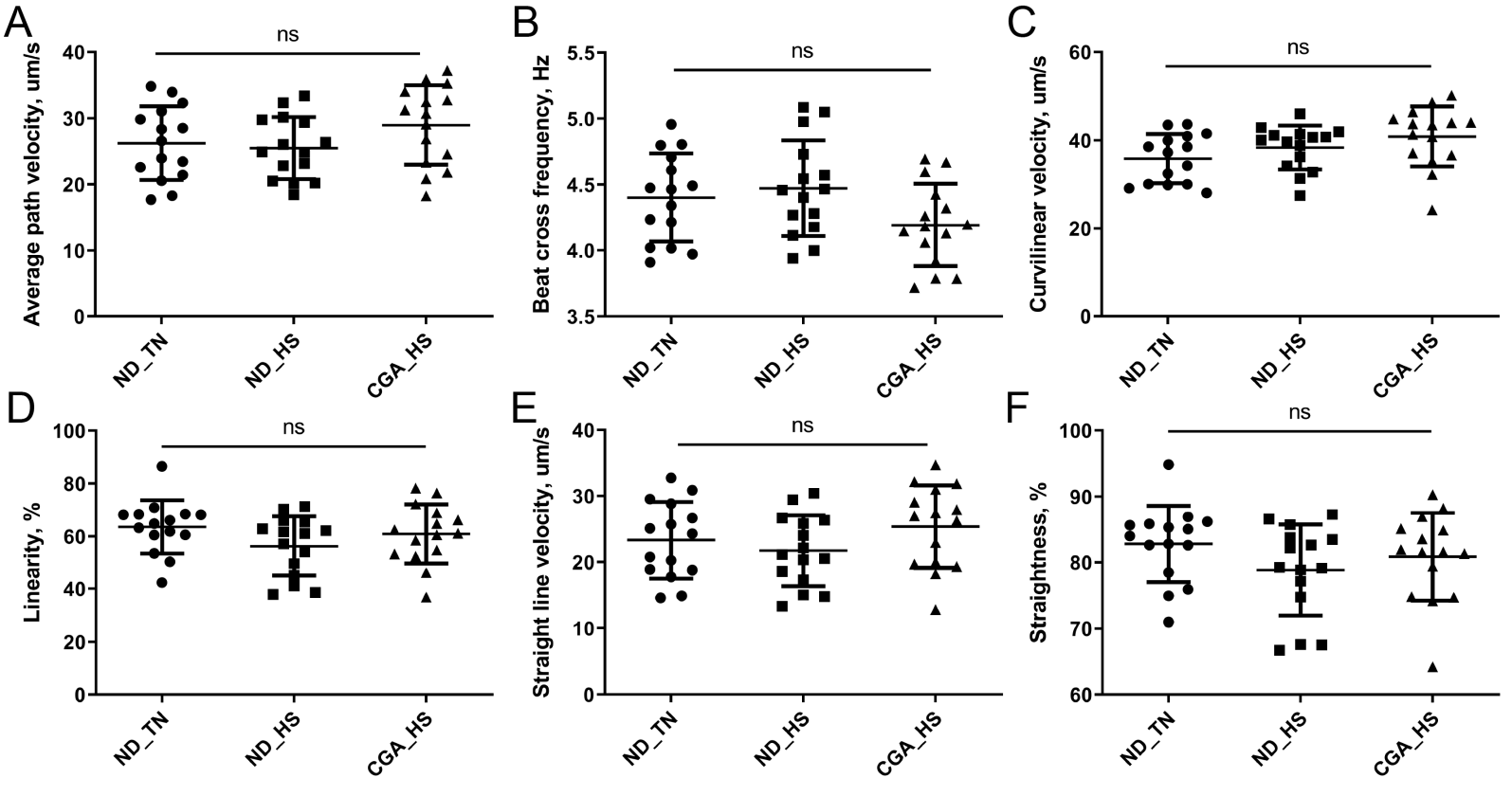


**Fig. S1** Effect of HS and CGA on kinetic parameters of boar sperm. **A** Average path velocity among different groups (*n* = 5). **B** Beat cross frequency among different groups (*n* = 5). **C** Curvilinear velocity among different groups (*n* = 5). **D** Linearity among different groups (*n* = 5). **E** Straight line velocity among different groups (*n* = 5). **F** Straightness among different groups (*n* = 5). Comparisons among multiple groups were performed using one-way ANOVA followed by Tukey’s multiple comparisons test. Data are expressed as the mean ± SD. Statistical significance was defined as *P* > 0.05 (ns) indicating no significant differences


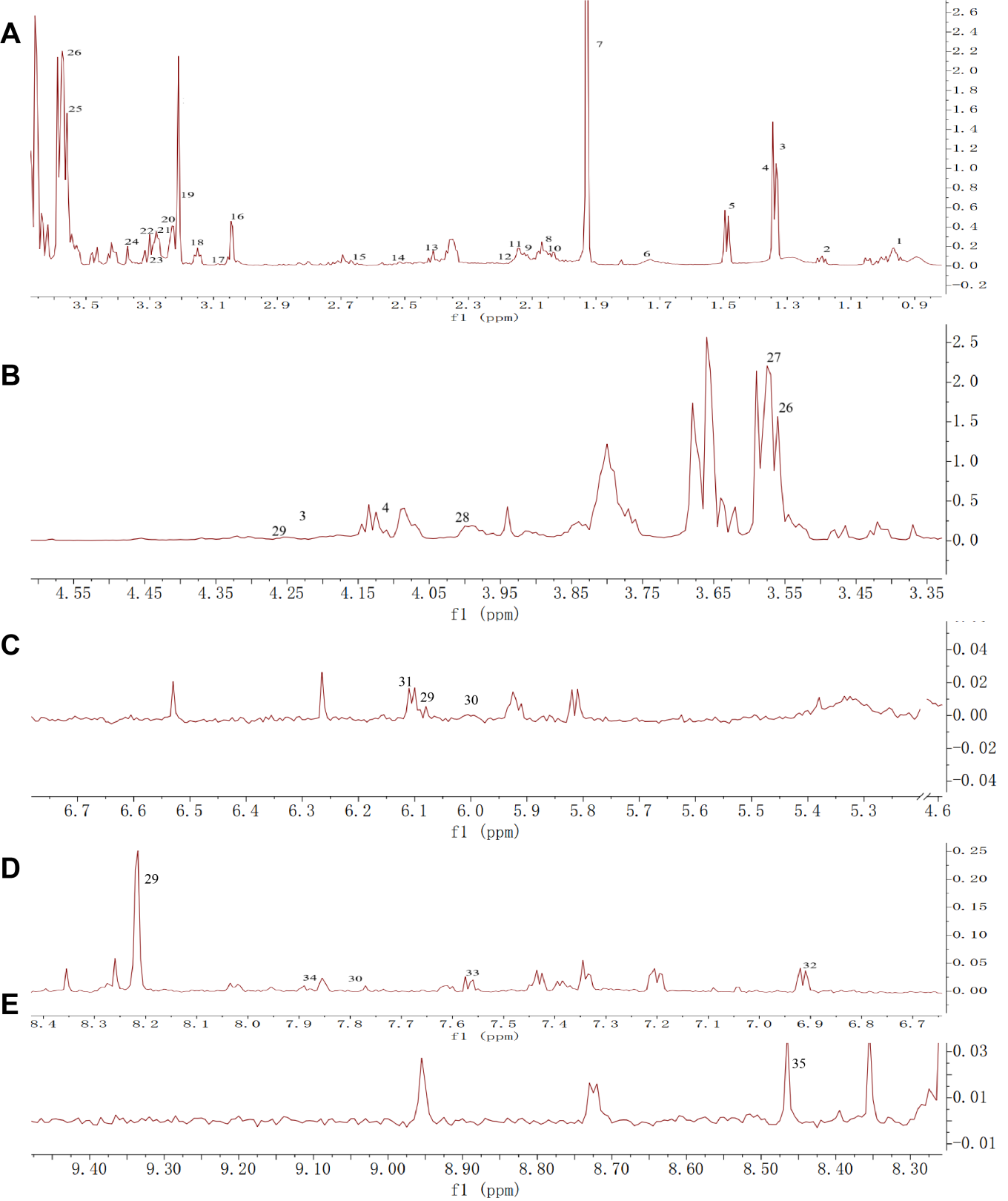


**Fig. S2** Representative 600 MHz ^1^H-NMR spectrum and serial numbers of labelled identified metabolites. **A** ¹H-NMR spectrum with range of 0.9-3.5 ppm. **B** ¹H-NMR spectrum with range of 2.25-4.55 ppm. **C** ¹H-NMR spectrum with range of 4.6-6.7 ppm. **D** ¹H-NMR spectrum with range of 6.7-8.4 ppm. ¹H NMR spectrum with range of 6.7-8.4 ppm. **E** ¹H-NMR spectrum with range of 8.3-9.4 ppm. The metabolites numbered from 1 to 35 are as follows:1-5: Branched chain amino acid, 3-hydroxybutyrate, threonine, lactate, alanine. 6-10: Lysine, acetate, proline, glutamate, glutamine. 11-15: Glutathione, methionine, succinate, citrate, aspartate. 16-20: Creatine, phenylalanine, ethanolamine, phosphocholine, choline. 21-25: Taurine, betaine, myo inositol, scyllo inositole, glycerol. 26-30: Glycine, phosphotidyl choline, ascorbate, adenosine/inosine, cytidine. 31-35: Adenosine/AMP, tyrosine, nicotinamide, xanthine, formate


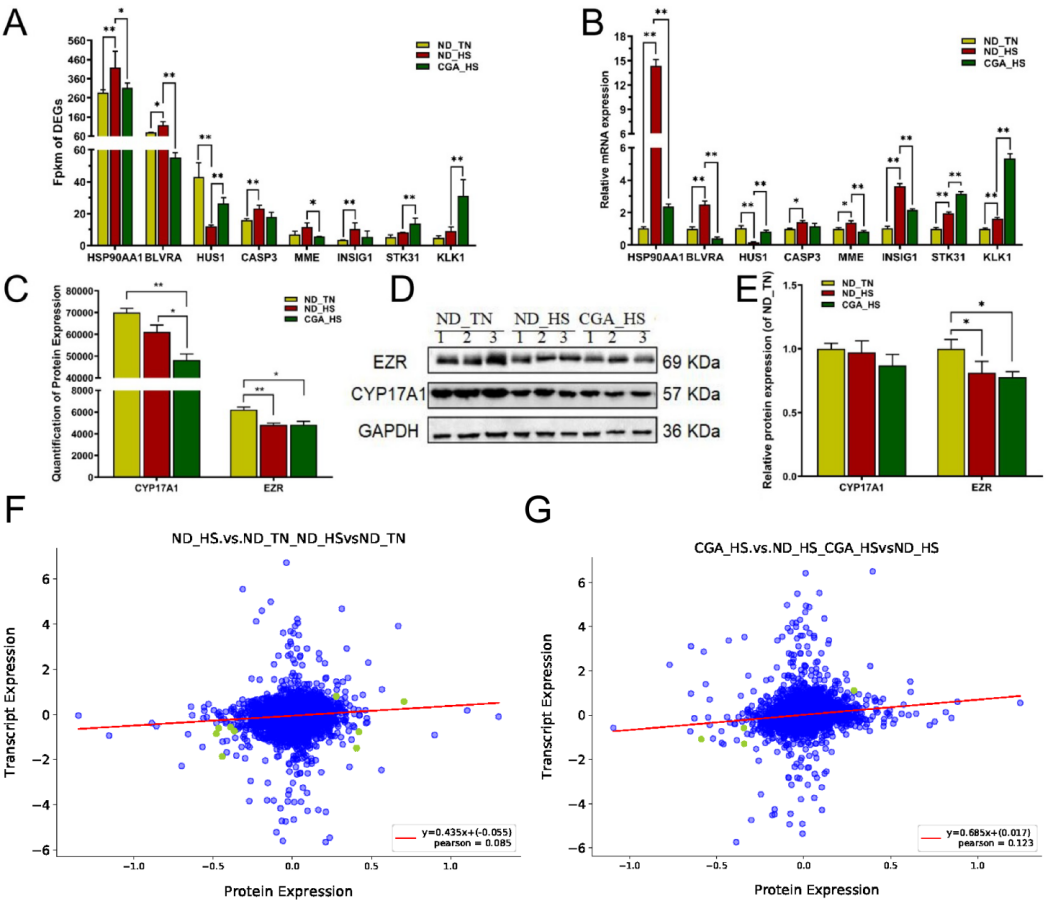


**Fig. S3** Validation of RNA-Seq and proteomics sequencing results and correlation analysis of transcriptome and proteome expression profiles. **A** The FPKM of 8 genes in transcriptome sequencing (*n* = 3). **B** The relative expression levels of genes in RT-qPCR (*n* = 3). **C** The quantitative values of EZR and CYP17A1 in proteome sequencing (*n* = 3). **D** The Western Blot of EZR and CYP17A1 in different samples. **E** The gray-scale analysis of EZR and CYP17A1 in different samples (*n* = 3). **F** and **F** Correlation analysis of transcriptome and proteome expression levels of ND_HS vs. ND_TN and CGA_HS vs. ND_HS. Each point in the figure represents a protein. Green points represent proteins with significantly different expression levels, while blue points represent those with no significant differences in expression. The *x*-axis shows the log_2_ (fold change) of the corresponding proteins in proteomic data, and the *y*-axis represents the log_2_ (fold change) of the corresponding genes in transcriptomic data. Comparisons among multiple groups were performed using one-way ANOVA followed by Tukey’s multiple comparisons test. Data are expressed as the mean ± SD. Statistical significance was defined as *P* < 0.05 (*) and *P* < 0.01 (**) indicating significant and highly significant differences between two groups, respectively
