# Supplementary material for: Multiomics analysis reveals that chlorogenic acid alleviates heat stress-induced oxidative damage in prepubertal boar testes via the BLVRA-GPX3 pathway: in vivo and in vitro evidence
Source: J Anim Sci Biotechnol. 2026 Jan 13;17:7. doi: 10.1186/s40104-025-01336-0 (PMC12798073; doi:10.1186/s40104-025-01336-0)
Supplement: Supplementary file 2 — Additional file 2: Table S1 Target sequence of BLVRA siRNA. Table S2 Primers used for RT-qPCR analysis. Table S3 Effects of CGA and HS on the growth performance of prepubertal boars. Table S4 Average THI during the heat treatment trial. Table S5 Chemical shift assignments of the metabolites observed in the 1H NMR spectra of aqueous extract from prepubertal porcine testicles. Table S6 Relative content of the aqueous metabolites in ND_TN, ND_HS and CGA_HS group. [file 40104_2025_1336_MOESM2_ESM.docx]

Table S1. Target sequence of *BLVRA* siRNA

| siRNA Name | Denote as | Target Sequence (5′–3′) |
| --- | --- | --- |
| si-ssc-BLVRA_1 | si-1 | CGAGTTCCTGAACCTGATT |
| si-ssc-BLVRA_2 | si-2 | GAGCACGTTGAACTCTTGA |
| si-ssc-BLVRA_3 | si-3 | CAGGCAGTTCCTTAACGCT |

Note: ssc: sus scrofa.

Table S2. Primers used for RT-qPCR analysis

| Symbol | Primer | Primer Sequence (5′–3′) | Gene ID (NCBI) |
| --- | --- | --- | --- |
| *BLVRA* | F-Primer  R-Primer | GCCACGAGGACTACATCAG  CCCAAAGAGGGAGACCAG | 100515289 |
| *CASP3* | F-Primer  R-Primer | TTTGCGTGCTTCTAAGCCAT  GGCAGGCCTGAATTATGAAA | 397244 |
| *INSIG1* | F-Primer  R-Primer | CCCCGAGGAGGTTATTGC  GGTTCTCCAAGGTGGCTGT | 403162 |
| *HSP90AA1* | F-Primer  R-Primer | ATCGCCCAGTTGATGTCGTT  GTGAGGGTCCGGTCTTGCT | 397028 |
| *HUS1* | F-Primer  R-Primer | GAGCTGGAACAGGAGAAC  ATGCGATTACTGCTTGAC | 100192318 |
| *GPX3* | F-Primer  R -Primer | CCCTTTGGCCTCGTCATTCT  GCTGGAAATTGGGGATGAAGC | 396598 |
| *GAPDH* | F-Primer  R -Primer | TCAAATGGGGTGATGCTGGT  GCAGAAGGGGCAGAGATGAT | 396823 |
| *KLK1* | F-Primer  R-Primer | GGACTACAGCCACGACCTCATGCTGC  GTCGGGGAATTCGAAGTCGTCTGG | 431673 |
| *MME* | F-Primer  R-Primer | AGCCTTCCCACAGCATTT  AGCCTCAGCCGAACCTAC | 100511536 |
| *STK31* | F-Primer  R-Primer | TGGCTTATCTGATGGTCC  GATGAAGTGCTCCGTGAA | 100524057 |

Table S3 Effects of CGA and HS on the growth performance of prepubertal boars

|  | ND_TN  (n = 10) | ND_HS  (n = 10) | CGA_HS  (n = 10) | SD | SEM | *P*-value |
| --- | --- | --- | --- | --- | --- | --- |
| **D1-D28** |  |  |  |  |  |  |
| Initial body weights (IBW), kg | 9.39 | 9.20 | 10.7 | 1.57 | 0.29 | 0.531 |
| Final body weights (FBW), kg | 19.56 ^b^ | 19.85 ^b^ | 24.05 ^a^ | 3.00 | 0.55 | 0.008 |
| Weight gain, (IBW-FBW), kg | 10.17 ^b^ | 10.65 ^b^ | 13.35 ^a^ | 2.54 | 0.46 | 0.013 |
| ADG, g | 363.10 ^b^ | 380.36 ^b^ | 476.79 ^a^ | 90.86 | 16.59 | 0.013 |
| ADFI, g/d | 601 ^b^ | 621 ^a^ | 629 ^a^ | 14.13 | 2.58 | 0.001 |
| F/G | 1.70 ^a^ | 1.69 ^a^ | 1.37 ^b^ | 0.34 | 0.06 | 0.044 |
| **D29-D35** |  |  |  |  |  |  |
| Initial body weights (IBW), kg | 19.56 ^b^ | 19.85 ^b^ | 24.05 ^a^ | 3.00 | 0.55 | 0.008 |
| Final body weights (FBW), kg | 23.00 ^b^ | 22.00 ^b^ | 26.00 ^a^ | 2.98 | 0.54 | 0.003 |
| Weight gain, (IBW-FBW), kg | 3.44 ^a^ | 2.15 ^c^ | 2.86 ^b^ | 0.69 | 1,23 | < 0.001 |
| ADG, g | 492.06 ^a^ | 307.14 ^c^ | 409.09 ^b^ | 98.55 | 17.99 | < 0.001 |
| ADFI, g/d | 714.29 ^a^ | 550 ^c^ | 657.14 ^b^ | 69.71 | 12.73 | < 0.001 |
| F/G | 1.50 ^b^ | 1.84 ^a^ | 1.65 ^ab^ | 0.29 | 0.05 | 0.020 |
| **D1-D35** |  |  |  |  |  |  |
| Initial body weights (IBW), kg | 9.39 | 9.20 | 10.70 | 1.57 | 0.29 | 0.531 |
| Final body weights (FBW), kg | 23.00 ^b^ | 22.00 ^b^ | 26.00 ^a^ | 2.98 | 0.54 | 0.003 |
| Weight gain, (IBW-FBW), kg | 13.61 ^b^ | 12.80 ^b^ | 15.95 ^a^ | 2.51 | 0.46 | 0.006 |
| ADG, g | 388.89 ^b^ | 365.71^b^ | 455.84 ^a^ | 71.83 | 13.11 | 0.006 |
| ADFI, g/d | 623.56 ^b^ | 606.80 ^c^ | 634.60 ^a^ | 13.04 | 2.38 | < 0.001 |
| F/G | 1.62 ^a^ | 1.69 ^a^ | 1.40 ^b^ | 1.57 | 0.25 | 0.022 |

Note: ADG: Average daily gain, ADFI: Average daily feed intake, F/G: ADFI/ADG, ND_TN: Normal diet with thermoneutral group, ND_HS: Normal diet with HS group, CGA_HS: CGA with HS group.

a,b,c Means in the same row with different superscripts differ significantly (*P* < 0.05).

Table S4 Average THI during the heat treatment trial

|  | ND_TN | ND_HS | CGA_HS | SD | SEM | *P*-value |
| --- | --- | --- | --- | --- | --- | --- |
| 8:00 | 69.74 | 70.16 | 70.84 | 1.44 | 0.31 | 0.37 |
| 13:00 | 69.67 ^b^ | 85.91 ^a^ | 86.37 ^a^ | 8.10 | 1.77 | < 0.001 |
| 18:00 | 68.87 ^b^ | 75.63 ^a^ | 76.09 ^a^ | 3.57 | 0.78 | < 0.001 |
| Average | 69.43 ^b^ | 77.21 ^a^ | 77.74 ^a^ | 3.93 | 0.86 | < 0.001 |

Note: THI: Temperature-humidity index. ND_TN: Normal diet with thermoneutral group (n = 10), ND_HS: Normal diet with HS group (n = 10), CGA_HS: CGA with HS group (n = 10).

a,b,c Means in the same row with different superscripts differ significantly (*P* < 0.05).

Table S5. Chemical shift assignments of the metabolites observed in the ^1^H NMR spectra of aqueous extract from prepubertal porcine testicles

| No | Metabolites | Ppm, δ^1^H multiplicity, | Bins used for quantification |
| --- | --- | --- | --- |
| 1 | Branched chain amino acid | 0.94t | 0.800-1.100 |
| 2 | 3-hydroxybutyrate | 1.19d (*J*=6.6 Hz) | 1.17-1.21 |
| 3 | Threonine | 1.31d, 4.25m | 1.30-1.31, 4.22-4.27 |
| 4 | Lactate | 1.34d (*J*=7.2 Hz), 4.13q (α-CH，β-CH_3_) | 1.32-1.36, 4.105-4.150 |
| 5 | Alanine | 1.47d (*J*=7.2 Hz), 3.79q (β-CH3) | 1.47-1.505, 3.79-3.83 |
| 6 | Lysine | 1.73m | 1.66-1.78 |
| 7 | Acetate | 1.93s (CH_3_) | 1.91-1.95 |
| 8 | Proline | 1.99m, 2.06m, 4.1m | 1.98-2.00, 2.045-2.06, 4.06-4.10 |
| 9 | Glutamate | 2.06m | 2.02-2.07 |
| 10 | Glutamine | 2.08m (β-CH_2_) | 2.065-2.085 |
| 11 | Glutathione | 2.15q, 3.77m | 2.11-2.15, 3.75-3.785 |
| 12 | Methionine | 2.16m (α-CH) | 2.155-2.165 |
| 13 | Succinate | 2.41s | 2.40-2.42 |
| 14 | Citrate | 2.52d, 2.62d | 2.505-2.58, 2.64-2.675 |
| 15 | Aspartate | 2.67dd | 2.68-2.70 |
| 16 | Creatine | 3.04s (CH_3_) | 3.03-3.05 |
| 17 | Phenylalanine | 3.11dd, 3.27dd | 3.10-3.12, 3.265-3.295, 3.88-3.92, 7.33-7.45 |
| 18 | Ethanolamine | 3.12m, 3,82m | 3.13-3.17, 3.825-3.86 |
| 19 | Phosphocholine | 3.20s (N(CH_3_)), 3.58m | 3.185-3.195, 3.55-3.60 |
| 20 | Choline | 3.21s (N(CH_3_)), 3.52m, 4.04m | 3.20-3.22, 3.51-3.545, 4.015-4.045 |
| 21 | Taurine | 3.23t, 3.42t | 3.225-3.25, 3.40-3.44 |
| 22 | Betaine | 3.27s, 3.94s | 3.265-3.275, 3.925-3.95 |
| 23 | Myo inositol | 3.28t, 3.56dd, 3.63dd | 3.25-3.295, 3.565-3.605, 3.61-3.645 |
| 24 | Scyllo inositole | 3.35s | 3.35-3.38 |
| 25 | Glycerol | 3.54dd, 3.63dd | 3.55-3.60, 3.61-3.645 |
| 26 | Glycine | 3.56s (CH_2_) | 3.55-3.565 |
| 27 | Phosphotidyl choline | 3.58m | 3.55-3.60 |
| 28 | Ascorbate | 4.0m, 4.49d | 3.97-4.01, 4.49-4.51 |
| 29 | Adenosine/inosine | 4.26m, 4.42m (*J* = 4.2 Hz), 6.08d, 8.21s | 4.27-4.34, 4.42-4.48, 6.085-6.12, 8.20-8.215 |
| 30 | Cytidine | 6.04d (*J*=7.8 Hz), 7.82d | 6.02-6.05, 7.795-7.83 |
| 31 | Adenosine/AMP | 6.10d, 8.24s, 8.35s | 6.085-6.12, 8.24-8.27, 8.34-8.35 |
| 32 | Tyrosine | 6.90d (*J*=8.4 Hz, 3/5-CH), 7.20d (*J* =8.4 Hz) | 6.90-6.93, 7.18-7.22 |
| 33 | Nicotinamide | 7.58m, 8.69dd | 7.545-7.585；8.70-8.74 |
| 34 | Xanthine | 7.9s | 7.885-7.905 |
| 35 | Formate | 8.46s (CH) | 8.45-8.475 |

Note: The type of splitting, that is, singlet as s, doublet as d, triplet as t, quadruplet as qd, multiplet as m; The functional groups in the molecules identified within the brackets that are associated with specific absorption and/or coupling constants *J*, and the protons involved are underlined.

Table S6 Relative content of the aqueous metabolites in ND_TN, ND_HS and CGA_HS group

| No | Metabolites | ND_TN | ND_HS | CGA_HS | SD | SEM | *P*-value |
| --- | --- | --- | --- | --- | --- | --- | --- |
| 1 | Branched chain amino acid | 3.32 | 2.94 | 2.66 | 0.48 | 0.12 | 0.190 |
| 2 | 3-hydroxybutyrate | 0.37 | 0.42 | 0.45 | 0.60 | 0.15 | 0.072 |
| 3 | Threonine | 5.41 | 4.95 | 4.67 | 0.25 | 0.07 | 0.081 |
| 4 | Lactate | 0.34 | 0.33 | 0.25 | 2.23 | 0.58 | 0.291 |
| 5 | Alanine | 5.69 | 6.29 | 5.93 | 0.18 | 0.05 | 0.507 |
| 6 | Lysine | 0.84 | 0.97 | 0.82 | 1.02 | 0.26 | 0.524 |
| 7 | Acetate | 8.98 ^b^ | 12.52 ^ab^ | 13.34 ^a^ | 0.04 | 0.01 | 0.026 |
| 8 | Proline | 2.27 | 2.25 | 2.23 | 0.96 | 0.43 | 0.829 |
| 9 | Glutamate | 2.09 ^a^ | 1.71 ^b^ | 1.87 ^ab^ | 0.37 | 0.10 | 0.041 |
| 10 | Glutamine | 1.10 ^a^ | 0.89 ^b^ | 0.99 ^ab^ | 1.31 | 0.34 | 0.029 |
| 11 | Glutathione | 2.98 ^a^ | 2.58 ^b^ | 2.62 ^ab^ | 0.15 | 0.04 | 0.040 |
| 12 | Methionine | 1.21 | 0.17 | 0.17 | 0.15 | 0.04 | 0.253 |
| 13 | Succinate | 0.29 | 0.28 | 0.23 | 0.00 | 0.00 | 0.058 |
| 14 | Citrate | 0.50 | 0.52 | 0.45 | 0.03 | 0.01 | 0.140 |
| 15 | Aspartate | 1.68 | 1.51 | 1.35 | 0.10 | 0.02 | 0.123 |
| 16 | Creatine | 0.86 | 0.79 | 0.78 | 0.02 | 0.01 | 0.202 |
| 17 | Phenylalanine | 2.53 | 2.26 | 2.13 | 0.01 | 0.00 | 0.092 |
| 18 | Ethanolamine | 2.60 | 2.34 | 2.07 | 0.01 | 0.00 | 0.108 |
| 19 | Phosphocholine | 8.39 | 9.81 | 10.15 | 0.60 | 0.15 | 0.265 |
| 20 | Choline | 4.47 | 4.12 | 3.87 | 0.48 | 0.12 | 0.149 |
| 21 | Taurine | 2.05 | 2.04 | 2.01 | 2.23 | 0.58 | 0.835 |
| 22 | Betaine | 1.26 | 1.11 | 1.11 | 0.25 | 0.07 | 0.251 |
| 23 | Myo inositol | 12.32 | 11.73 | 12.00 | 0.18 | 0.05 | 0.417 |
| 24 | Scyllo inositole | 0.44 | 0.45 | 0.47 | 1.02 | 0.26 | 0.277 |
| 25 | Glycerol | 12.53 | 12.04 | 12.42 | 0.04 | 0.01 | 0.598 |
| 26 | Glycine | 2.72 ^ab^ | 2.49 ^b^ | 3.00 ^a^ | 0.96 | 0.43 | 0.031 |
| 27 | Phosphotidyl choline | 10.36 | 9.71 | 10.04 | 0.37 | 0.10 | 0.496 |
| 28 | Ascorbate | 0.89 | 1.02 | 1.05 | 1.31 | 0.34 | 0.116 |
| 29 | Adenosine/inosine | 0.82 | 0.83 | 0.67 | 0.15 | 0.04 | 0.115 |
| 30 | Cytidine | 0.0073 ^ab^ | 0.0036 ^b^ | 0.0085 ^a^ | 0.15 | 0.04 | 0.019 |
| 31 | Adenosine/AMP | 0.08 | 0.09 | 0.07 | 0.003 | 0.001 | 0.375 |
| 32 | Tyrosine | 0.40 | 0.35 | 0.27 | 0.03 | 0.01 | 0.054 |
| 33 | Nicotinamide | 0.13 ^a^ | 0.13 ^a^ | 0.10 ^b^ | 0.10 | 0.02 | 0.029 |
| 34 | Xanthine | 0.02 | 0.02 | 0.02 | 0.02 | 0.01 | 0.089 |
| 35 | Formate | 0.04 | 0.04 | 0.03 | 0.01 | 0.00 | 0.22 |

Note: ND_TN: Normal diet with thermoneutral group (n = 5), ND_HS: Normal diet with HS group (n = 5), CGA_HS: CGA with HS group (n = 5).

a,b Means in the same row with different superscripts differ significantly (*P* < 0.05).
