# Supplementary material for: Multiomics analysis reveals that chlorogenic acid alleviates heat stress-induced oxidative damage in prepubertal boar testes via the BLVRA-GPX3 pathway: in vivo and in vitro evidence
Source: J Anim Sci Biotechnol. 2026 Jan 13;17:7. doi: 10.1186/s40104-025-01336-0 (PMC12798073; doi:10.1186/s40104-025-01336-0)
Supplement: Supplementary file 8 — Additional file 8: The full uncropped Western blots images. [file 40104_2025_1336_MOESM8_ESM.docx]

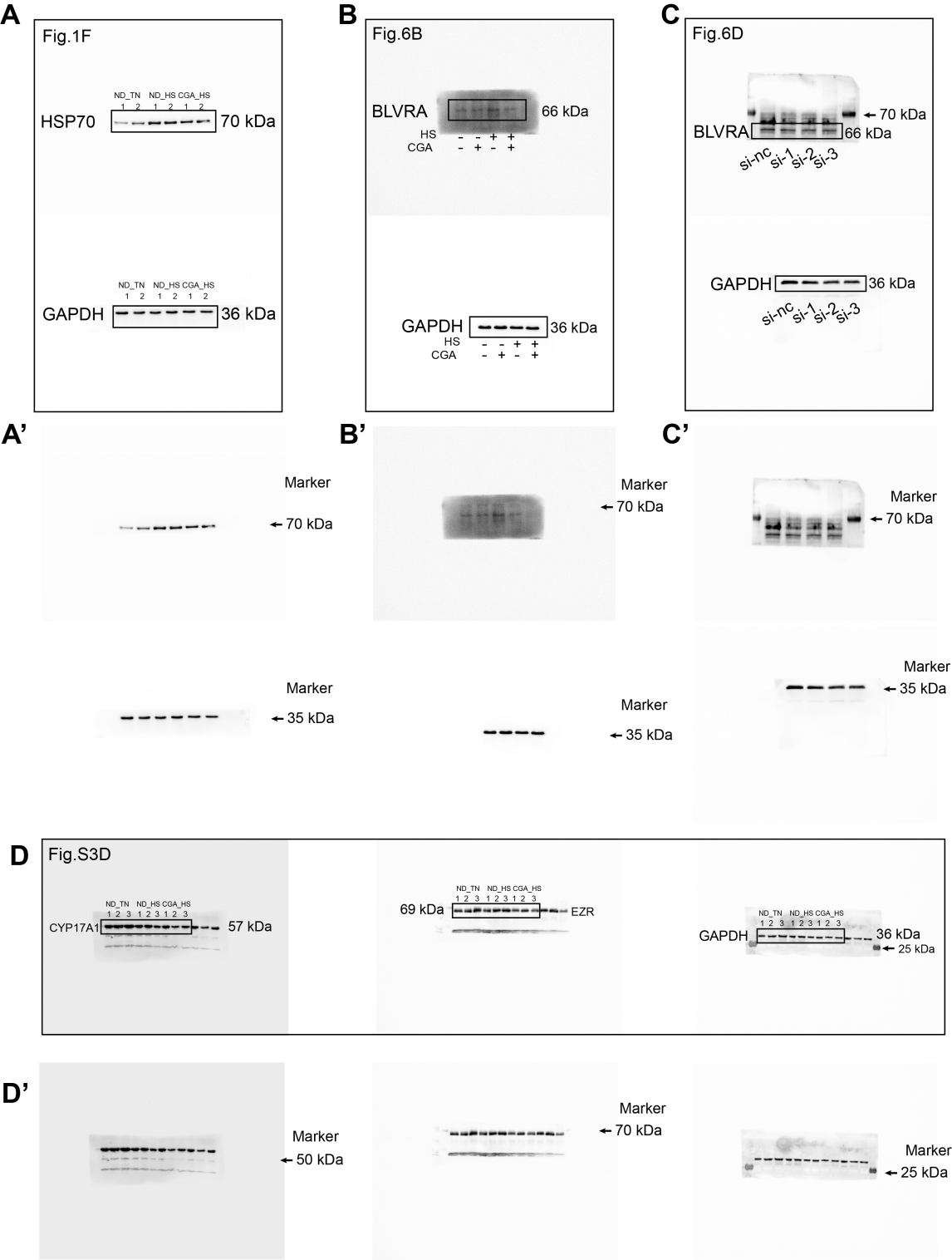


**Additional file 8** The full uncropped Western blot images. **A** Full uncropped Western blots images for Fig. 1F. **B** Full uncropped Western blots images for Fig. 6B. **C** Full uncropped Western blots images for Fig. 6D. **D** Full uncropped Western blots images for Fig. S3D. **A**’–**D**’ The corresponding pictures with markers labeled
